# Scalable process discovery and conformance checking

**DOI:** 10.1007/s10270-016-0545-x

**Published:** 2016-07-08

**Authors:** Sander J. J. Leemans, Dirk Fahland, Wil M. P. van der Aalst

**Affiliations:** Eindhoven University of Technology, Eindhoven, The Netherlands

**Keywords:** Big data, Scalable process mining, Block-structured process discovery, Directly-follows graphs, Algorithm evaluation, Rediscoverability, Conformance checking

## Abstract

Considerable amounts of data, including process events, are collected and stored by organisations nowadays. Discovering a process model from such event data and verification of the quality of discovered models are important steps in process mining. Many discovery techniques have been proposed, but none of them combines scalability with strong quality guarantees. We would like such techniques to handle billions of events or thousands of activities, to produce sound models (without deadlocks and other anomalies), and to guarantee that the underlying process can be rediscovered when sufficient information is available. In this paper, we introduce a framework for process discovery that ensures these properties while passing over the log only once and introduce three algorithms using the framework. To measure the quality of discovered models for such large logs, we introduce a model–model and model–log comparison framework that applies a divide-and-conquer strategy to measure recall, fitness, and precision. We experimentally show that these discovery and measuring techniques sacrifice little compared to other algorithms, while gaining the ability to cope with event logs of 100,000,000 traces and processes of 10,000 activities on a standard computer.

## Introduction

Considerable amounts of data are collected and stored by organisations nowadays. For instance, ERP systems log business transaction events, high-tech systems such as X-ray machines record software and hardware events, and web servers log page visits. Typically, each action of a user executed with the system, e.g. a customer filling in a form or a machine being switched on, can be recorded by the system as an event; all events related to the same process execution, e.g. a customer order or an X-ray diagnosis, are grouped in a *trace* (ordered by their time); an *event log* contains all recorded traces of the system. Process mining aims to extract information from such event logs, for instance social networks, business process models, compliance to rules and regulations, and performance information (e.g. bottlenecks) [46].

**Fig. 1:**
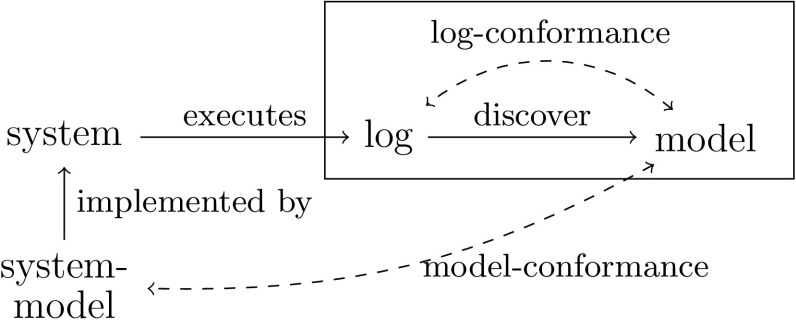
Process discovery and conformance checking in their context. The *box* contains a typical process mining project’s scope

In this paper, we focus on two process mining challenges: process discovery and conformance checking. Figure 1 shows the context of these two challenges: a real-life business process (a *system*) is running, and the executed process steps are recorded in an event log. In *process discovery*, one assumes that the inner workings of the system are unknown to the analyst and cannot be obtained otherwise. Therefore, process discovery aims to learn a process model from an event log, which describes the system as it actually happened (in contrast to what is assumed to have happened) [50]. Two main challenges exist in process discovery: first, one would like to learn an easy-to-understand model that captures the actual behaviour. Second, the model should have a proper formal interpretation, i.e. have well-defined behavioural semantics and be free of deadlocks and other anomalies (be *sound*) [28]. In Sect. 2, we explore these challenges in more detail and explore how they are realised in existing algorithms and settings. Few existing algorithms solve both challenges together.

In contrast, *conformance checking* studies the differences between a process model and reality. We distinguish two types of conformance checking. First, the model can be compared with a log. Such log conformance checking can provide insight into the real behaviour of an organisation, by highlighting which traces deviate from the model, and where in the model deviations occur [50]. Second, the model can be compared with a model of the system (but only if such a model is available). Model conformance checking can be used to highlight differences between different snapshots of a process or to verify that a process model conforms to a design made earlier [19]. Moreover, model conformance checking can be used to evaluate discovery algorithms by choosing a system model and quantifying the similarity between this chosen model and the models discovered by discovery algorithms. In Sect. 2 we discuss both log and model conformance checking in more detail.

*Large event logs* Current process discovery and conformance checking techniques work reasonably well on smaller event logs, but might have difficulties handling larger event logs. Commercial techniques such as Fluxicon Disco [23] and Celonis Process Mining offer the scalability to handle larger event logs, but usually do not provide strong quality guarantees or do not support parallelism. For instance, discovery techniques typically require the event log to fit in main memory and require the log to be rather complete, i.e. most of the possible behaviour must be present. Reducing the log size to fit in memory, e.g. through sampling, may yield incomplete event logs, which for discovery may lead to overfitting models (showing only the behaviour of the sample but not of the system) or underfitting models (showing arbitrary behaviour beyond the sample log) (for discovery). For conformance checking, such logs may lead to skewed measurements [50].

**Fig. 2:**
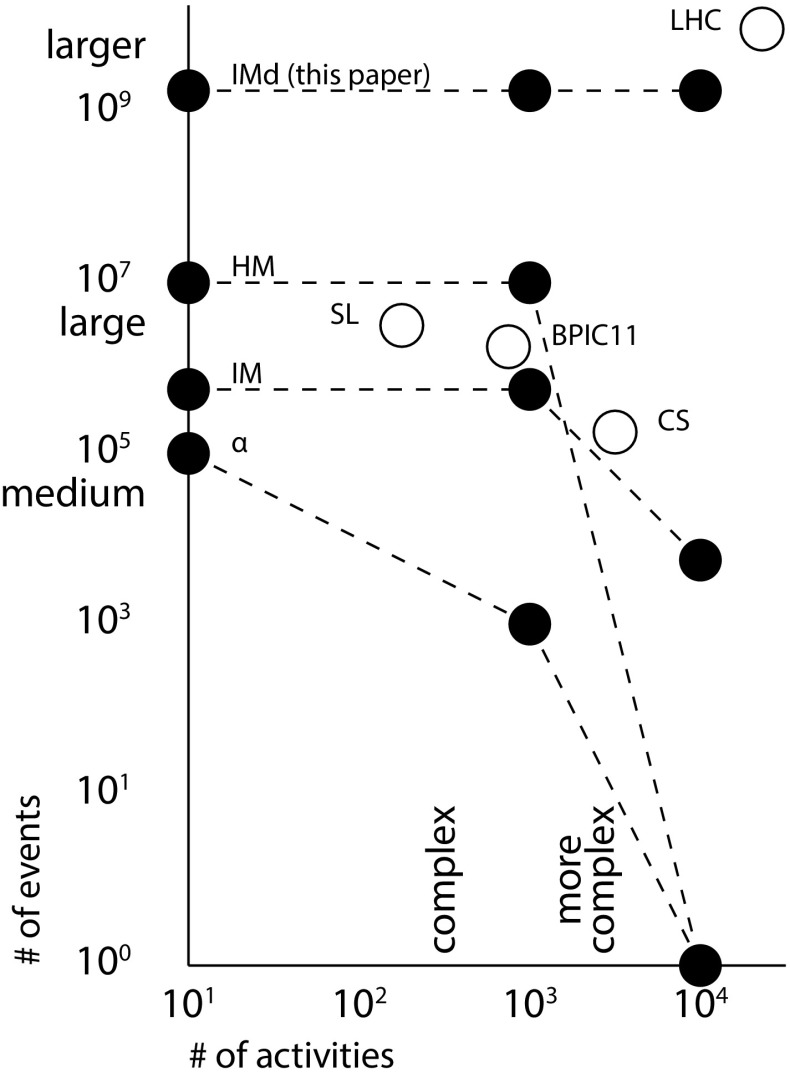
Scales used in this paper. The *dots* denote maximum number of events several discovery algorithms could handle (Sect. 6), for Inductive Miner (IM), the Heuristics Miner (HM) and the $\alpha$-algorithm ($\alpha$). These algorithms will be introduced in Sect. 2. The *circles* denote the mentioned logs

Event logs can be ‘big’ in two dimensions: many events and many activities (i.e. the different process steps). From our experiments (Sect. 6), we identified relevant gradations for these dimensions: for the number of activities we identified complex logs, i.e. containing hundreds of activities, and more complex logs, i.e. containing thousands of activities. For the number of events we identified medium logs, i.e. containing tens of thousands of events, large logs, i.e. containing millions of events, and larger logs, i.e. containing billions of events.

In our experiments we observed that existing process discovery algorithms with strong quality guarantees, e.g. soundness, can handle medium logs (see IM in Fig. 2; the algorithms will be introduced later). Algorithms not providing such guarantees (e.g. $\alpha$, HM) can handle large logs, but fail on larger logs. In the dimension of the number of different activities, experiments showed that most algorithms could not handle complex processes. Current conformance checking techniques in our experiments and [38] seem to be unable to handle medium *or* complex event logs.

Such numbers of events and activities might seem large for a complaint-handling process in an airline; however, processes of much larger complexity exist. For instance, even simple software tools contain hundreds or thousands of different methods. We obtained a large and complex log (SL) that will be used in the evaluation. To study or reverse engineer such software, studies [33] have recorded method calls in event logs (at various levels of granularity), and process mining and software mining techniques have been used on small examples to perform the analyses [33, 40]. complex logs can, for instance, be found in hospitals: the BPI Challenge log of 2011 (BPIC11) [56] was recorded in the emergency department of a Dutch hospital and contains over 600 activities [56]. Even though this log is just complex and medium, current discovery techniques have difficulties with this log (we will use it in the evaluation). Other areas in which more complex logs appear are click-stream data from websites, such as the website of a dot-com start-up, which produced an event log containing 3300 activities (CL) [26]. Even more difficult logs could be extracted from large machines, such as the Large Hadron Collider, in which over 25,000 distributed communicating components form just a part of the control systems [25], resulting in complicated behaviour that could be analysed using scalable process mining techniques. In the future, we aim to extract such logs and apply our techniques to it, but currently, we would only discover a model but would not be able to process the discovered model further (no conformance checking and no visualisation on that scale). Nevertheless, we will show in our evaluation that our discovery techniques are able to handle such logs.

*Problem definition and contribution* In this paper, we address two problems: applying process discovery to larger and more complex logs, and conformance checking to medium and complex logs. We introduce two scalable frameworks: one for process discovery, the *Inductive Miner—directly-follows framework* (IMd framework), and one for conformance checking: the *Projected Conformance Checking framework* (pcc framework). We instantiate these frameworks to obtain several algorithms, each with their specific purposes. For discovery, we show how to adapt an existing family of algorithms that offers several quality guarantees (the Inductive Miner framework (IM framework) [29]), such that is scales better and works on larger and more complex logs. We show that the recursion on event logs used by the IM framework can be replaced by recursion on an abstraction (i.e. the so-called *directly-follows graph* [29]), which can be computed in a single pass over the event log. We show that this principle can also be applied to incomplete event logs (when a discovery technique has to infer missing information) and logs with infrequent behaviour or noise (when a discovery algorithm has to identify and filter the events that would degrade model quality); we present corresponding algorithms. Incompleteness and infrequency/noise pose opposing challenges to discovery algorithms, i.e. not enough and too much information. For these purposes, we introduce different algorithms. For conformance checking, we introduce the configurable divide-and-conquer pcc framework to compare logs to models and models to models. Instead of comparing the complete behaviour over all activities, we decompose the problem into comparing behaviour for subsets of activities. For each such subset, a recall, fitness, or precision measure is computed. The averages over these subsets provide the final measures, while the subsets with low values give information about the location in the model/log/system-model where deviations occur.

*Results* We conducted a series of experiments to test how well algorithms handle large logs and complex processes. We found that the IMd framework provides the scalability to handle all kinds of logs up to larger and more complex logs (see Fig. 2). In a second series of experiments we investigated the ability of several discovery algorithms to rediscover the original system model: we experimented to analyse the influence of log sizes, i.e. completeness of the logs, to analyse the influence of noise, i.e. randomly appearing or missing events in process executions, and to assess the influence of infrequent behaviour, i.e. structural deviations from the system model during execution. We found that the new discovery algorithms perform comparable to existing algorithms in terms of model quality, while providing much better scalability. In a third experiment, we explored how the new discovery algorithms handle large and complex real-life logs.

In all of these experiments, the new pcc framework was applied to assess the quality of the discovered models with respect to the log and where applicable the system, as existing conformance checking techniques could not handle medium or complex logs and systems. We compared the new conformance checking techniques to existing techniques, and the results suggest that the new techniques might be able to replace existing less scalable techniques. In particular, model quality with respect to a log can now be assessed in situations where existing techniques fail: up to large and complex logs.

*Relation to earlier papers* This paper extends the work presented in [32]. We present the new pcc framework, which is able to cope with medium and complex logs and models. This allows us to analyse and compare the quality of the IMd framework to other algorithms on such event logs in detail.

*Outline* First, process mining is discussed in more detail in Sect. 2. Second, process trees, directly-follows graphs, and cuts are introduced in Sect. 3. In Sect. 4, the IMd framework and three algorithms using it are introduced. We introduce the pcc framework in Sect. 5. The algorithms are evaluated in Sect. 6 using this pcc framework. Section 7 concludes the paper.

## Process mining

In this section, we discuss conformance checking and process discovery in more detail.

### Conformance checking

The aim of conformance checking is to verify a process model against reality. As shown in Fig. 1, two types of conformance checking exist: log–model conformance checking and model–model conformance checking. In log–model conformance checking, reality is assumed to be represented by an event log, while in model–model conformance checking, a representation of the system is assumed to be present and to represent reality. Such a system is usually given as another process model, to which we refer to as *system model*.

#### Log–model conformance checking

To compare a process model to an event log, several quality measures have been proposed [2]. For instance, *fitness* expresses the part of the event log that is represented by the model, *log-precision* expresses the behaviour in the model that is present in the event log, *generalisation* expresses the likelihood that future behaviour will be representable by the model, and *simplicity* expresses the absence of complexity in a model [2] to represent its behaviour.

Several techniques and measures have been proposed to measure fitness and precision, such as token-based replay [43], alignments [1, 2], and many more: for an overview, see [60]. Some techniques that were proposed earlier, such as token-based replay [43], cannot handle non-determinism well, i.e. silent activities ($\tau$) and duplicate activities. Later techniques, such as alignments [2], can handle non-determinism by exhaustively searching for the model trace that has the least deviations from a given log trace (according to some cost function). However, even optimised implementations of these techniques cannot deal with medium or complex event logs and models [38]. To alleviate this problem, decomposition techniques have been proposed, using the general principles in [52], for instance using passages [47] or single-entry–single-exit decompositions [38]. The pcc framework uses the insights of [52] by checking conformance on small subsets of activities.

#### Model–model conformance checking

For a more elaborate overview of this field, we refer to [19] and [7].

Typically, the quality of a process discovery algorithm is measured using log conformance checking, i.e. a discovered model is compared to an event log. Alternatively, discovered model and system model could be compared directly. Ideally, both would be compared on branching bisimilarity or even stronger notions of equivalence [58], thereby taking the moments of choice into account. However, as an event log describes a language and does not contain information about choices, the discovered model will lack this information as well and we consider comparison based on languages (trace equivalence, a *language* is the set of traces of a log, or the set of traces that a model can produce).

One such technique is [5]. This approach translates the models into partially ordered runs annotated with exclusive relationships (event structures), which can be generated from process models as well [5]. However, event structures have difficulties supporting loops by their acyclic nature and constructing them requires a full state-space exploration.

As noted in [19], many model–model comparison techniques suffer from exponential complexity due to concurrency and loops in the models. To overcome this problem, several techniques apply an abstraction, for instance using causal footprints [50, 55], weak order relations [68], or behavioural profiles [27, 62]. Another technique to reduce the state space is decompose the model in pieces and perform the computations on these pieces individually [27]. Our approach (pcc framework, which applies to both log–model and model–model conformance checking) applies an abstraction using a different angle: we project on subsets of activities, thereby generalising over many of these abstractions, i.e. many relations between the projected activities are captured. Moreover, our approach handles any formalism of which the executable semantics can be described by deterministic finite automata (DFAs), which includes BPMN, UML-ADs, labelled Petri nets, and allows for models with duplicate activities, silent steps, and anomalies such as potential deadlocks.

### Process discovery

Process discovery aims at discovering a process model from an event log (see Fig. 1). We first sketch some challenges that discovery algorithms face, after which we discuss existing discovery approaches.

*Challenges* Several factors challenge process discovery algorithms. One such challenge is that the resulting process model should have well-defined behavioural semantics and be sound [50]. Even though an unsound process model or a model without a language, i.e. without a definition of traces the model expresses, might be useful for manual analysis, conformance checking and other automated techniques can obviously not provide accurate measures on such models [28, 59]. The IMd framework uses its representational bias to provide a language and to guarantee soundness: it discovers an abstract hierarchical view on workflow nets [50], *process trees*, that is guaranteed to be sound [11].

Another challenge of process discovery is that for many event logs the different measures, e.g. fitness, log-precision, generalisation and simplicity, are competing, i.e. there might not exist a model that scores well on all criteria [12]. Thus, discovery algorithms have to balance these measures, and this balance might depend on the use case at hand, e.g. auditing questions are best answered using a model with high fitness, optimisations are best performed on a model with high log-precision, implementations might require a model with high generalisation, and human interpretation is eased by a simple model [12].

A desirable property of discovery algorithms is having the ability to rediscover the language of the system (*rediscoverability*); we assume the system and the system model to have the same behaviour for rediscoverability. Rediscoverability is usually proven using assumptions on both system and event log: the system typically must be of a certain class, and the event log must contain enough correct information to describe the system well [29]. Therefore, three more challenges of process discovery algorithms are to handle (1) *noise* in the event log, i.e. random absence or presence of events [13], (2) *infrequent behaviour*, i.e. behaviour that occurs less frequent than ‘normal’ behaviour, i.e. the exceptional cases. For instance, most complaints sent to an airline are handled according to a model, but a few complaints are so complicated that they require ad hoc solutions. This behaviour could be of interest or not, which depends on the goal of the analysis [50]. (3) *incompleteness*, i.e. the event log does not contain ‘enough’ information. The notion of what ‘enough’ means depends on the discovery algorithm [6, 29]. Even though rediscoverability is desirable, it is a formal property, and it is not easy to compare algorithms using it. However, the pcc framework allows to perform experiments to quantify how rediscoverability is influenced by noise, infrequent behaviour, and incompleteness.

A last challenge arises from the main focus of this paper, i.e. highly scalable environments. Ideally, a discovery technique should linearly pass over the event log once, which removes the need to keep the event log in memory. In the remainder of this section, we discuss related process discovery techniques and their application in scalable environments.

*Sound process discovery algorithms* Process discovery techniques such as the *Evolutionary Tree Miner* (ETM) [11], the *Constructs Competition Miner* (CCM) [41], *Maximal Pattern Mining* (MPM) [34], and *Inductive Miner* (IM) [29] provide several quality guarantees, in particular soundness and some offer rediscoverability, but do not manage to discover a model in a single pass. ETM applies a genetic strategy, i.e. generates an initial population, and then applies random crossover steps, selects the ‘best’ individuals from the population and repeats. While ETM is very flexible towards the desired log measures to which respect the model should be ‘best’ and guarantees soundness, it requires multiple passes over the event log and does not provide rediscoverability.

CCM and IM use a divide-and-conquer strategy on event logs. In the Inductive Miner framework (IM framework), first an appropriate cut of the process activities is selected; second, that cut is used to split the event log into sublogs; third, these sublogs are recursed on, until a base case is encountered. If no appropriate cut can be found, a fall-through (‘anything can happen’) is returned. CCM works similarly by having several process constructs compete with one another. While both CCM and the IM framework guarantee soundness and IM guarantees rediscoverability (for the class of models described in Appendix 1), both require multiple passes through the event log (the event log is being split and recursed on).

MPM first constructs a prefix tree of the event log. Second, it folds leaves to obtain a process model, thereby applying local generalisations to detect concurrency. The MPM technique guarantees soundness and fitness, allows for noise filtering and can reach high precision, but it does so at the cost of simplicity: typically, lots of activities are duplicated. Inherently, the MPM technique requires random access to the event log and a single pass does not suffice.

*Other process discovery algorithms* Other process discovery techniques are, for instance, the $\alpha$ *-algorithm* ($\alpha$) and its derivatives [49, 65, 66], the *Heuristics Miner* [64] (HM), the *Integer Linear Programming* miner [54] (ILP) and several commercial tools, such as *Fluxicon Disco* (FD) [23] and *Perceptive Process Mining* (two versions: PM1 and PM2).

**Fig. 3:**
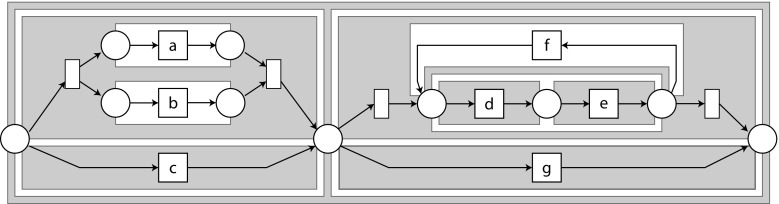
A block-structured hierarchical workflow net; the block structure is denoted by filled regions (image taken from [30])

Some of these guarantee soundness, but do not support explicit concurrency (FD, PM1) [31]. The ILP miner guarantees fitness and can guarantee that the model is empty after completion, but only for the traces seen in the event log, i.e. the models produced by ILP are usually not sound. However, most of these algorithms ($\alpha$, HM, ILP, PM2) neither guarantee soundness nor even provide a final marking, which makes it difficult to determine their language (see Appendix 5), and thus, their models are difficult to analyse automatically (though, such unsound models can still be useful for manual analysis).

Several techniques (e.g. $\alpha$, HM) satisfy the single-pass requirement. These algorithms first obtain an abstraction from the log, which denotes what activities directly follow one another; in HM, this abstraction is filtered. Second, from this abstraction a process model is constructed. Both $\alpha$ and HM have been demonstrated to be applicable in highly scalable environments: event logs of 5 million traces have been processed using map-reduce techniques [21]. Moreover, $\alpha$ guarantees rediscoverability, but neither $\alpha$ nor HM guarantees soundness. We show that our approach offers the same scalability as HM and $\alpha$, but provides both soundness and rediscoverability.

Some commercial tools such as FD and PM1 offer high scalability, but do not support explicit concurrency [31]. Other discovery techniques such as the language-based region miner [9, 10] or the state-based region miner [17] guarantee fitness but neither soundness nor rediscoverability nor work in single pass.

*Software mining* In the field of software mining, similar techniques have been used to discover formal specifications of software. For instance, in [40] and [4], execution sequences of software runs (i.e. traces) are recorded in an event log, from which techniques extract, e.g., valid execution sequences on the methods of an API. Such valid execution sequences can then be used to generate documentation. Process discovery differs from software mining in focus and challenges: process discovery aims to find process models with soundness and concurrency and is challenged, e.g., by deviations from the model (noise, infrequent behaviour) and readability requirements of the discovered models, while for software mining techniques, the system is fixed and challenges arise from, e.g., nesting levels [40], programmed exceptions [67], and collaborating components [22].

*Streams* Another set of approaches that aims to handle even bigger logs assumes that the event log is an unbounded stream of events. Some approaches such as [18, 24] work on click-stream data, i.e. the sequence of web pages users visit, to extract, for instance, clusters of similar users or web pages. However, we aim to extract end-to-end process models, in particular containing parallelism. HM, $\alpha$, and CCM have been shown to be applicable in streaming environments [14, 42], and any single-pass discovery algorithm (thus the IMd framework as well) can be converted into a streaming algorithm, but will have to deal with the same discovery challenges as described before.

## Preliminaries

To overcome the limitations of process discovery on large event logs, we will combine the single-pass property of directly-follows graphs with a divide-and-conquer strategy. This section recalls these existing concepts. The new algorithms are introduced in Sect. 4.

### Basic notions

*Event logs* An *event log* is a multiset of *traces* that denote process executions. For instance, the event log $[\langle a, b, c \rangle , \langle b, d \rangle ^2]$ denotes the event log in which the trace consisting of the activity *a* followed by the activity *b* followed by the activity *c* was executed once, and the trace consisting of *b* followed by *d* was executed twice.

*Process trees* A *process tree* is an abstract representation of a block-structured hierarchical process model, in which the leaves represent the *activities*, i.e. the basic process steps, and the *operators* describe how their children are to be combined [11]. $\tau$ denotes the activity whose execution is not visible in the event log. We consider four operators: $\times$, $\rightarrow$, $\wedge$, and $\circlearrowleft$. $\times$ describes the exclusive choice between its children, $\rightarrow$ the sequential composition, and $\wedge$ the parallel composition. The first child of a loop $\circlearrowleft$ is the *body* of the loop, and all other children are *redo* children. First, the body must be executed, followed by zero or more iterations of a redo child and the body. A formal definition is given in Appendix 1; we give an example here: Fig. 3 shows the Petri net corresponding to the process tree $\rightarrow (\times (\wedge (a, b), c), \times (\circlearrowleft (\rightarrow (d, e), f), g))$. Process trees are inherently sound.

**Fig. 4:**
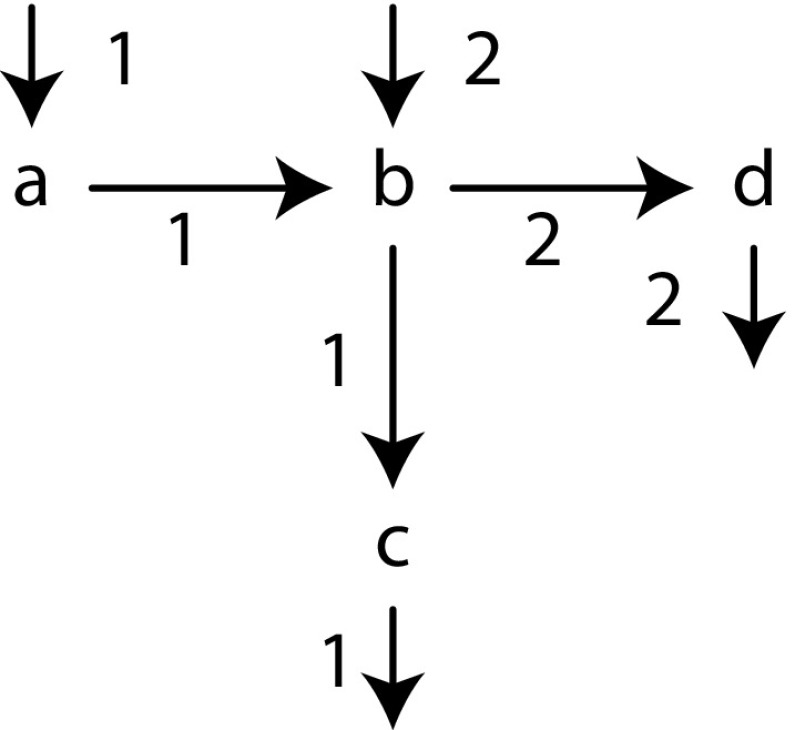
Example of a directly-follows graph

**Fig. 5:**
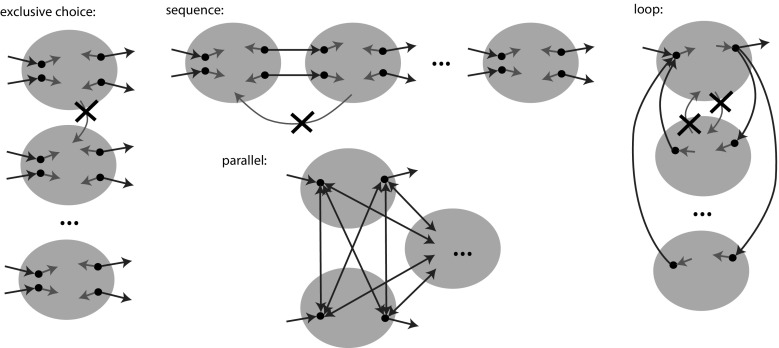
Cut characteristics

*Directly-follows graphs* A *directly-follows graph* can be derived from a log and describes what activities follow one another directly, and with which activities a trace starts or ends. In a directly-follows graph, there is an edge from an activity *a* to an activity *b* if *a* is followed directly by *b*. The weight of an edge denotes how often that happened. For instance, the directly-follows graph of our example log $[\langle a, b, c \rangle , \langle b, d \rangle ^2]$ is shown in Fig. 4. Note that the multiset of start activities is $[a, b^2]$ and the multiset of end activities is $[c, d^2]$. A directly-follows graph can be obtained in a single pass over the event log with minimal memory requirements [21].

*Cuts, characteristics, and the Inductive Miner framework.* A *partition* is a non-overlapping division of the activities of a directly-follows graph. For instance, $(\{a, b\}, \{c, d\})$ is a binary partition of the directly-follows graph in Fig. 4. A *cut* is a partition combined with a process tree operator, for instance $(\rightarrow , \{a, b\}, \{c, d\})$. In the IM framework, finding a cut is an essential step: its operator becomes the root of the process tree, and its partition determines how the log is split.

The IM framework [29] discovers the main cut and projects the given log onto the activity partition. In case of loops, each iteration becomes a new trace in the projected sublog. Subsequently, for each sublog its main cut is detected and recursion continues until reaching partitions with singleton elements; these become the leaves of the process tree. If no cut can be found, a generalising fall-through is returned that allows for any behaviour (a ‘flower model’). By the use of process trees, the IM framework guarantees sound models and makes it easy to guarantee fitness. The IM framework is formalised in Appendix 1.

Suppose that the log is produced by a process which can be represented by a process tree *T*. Then, the root of *T* leaves certain characteristics in the log and in the directly-follows graph. The most basic algorithm that uses the IM framework, i.e. IM [29], searches for a cut that matches these characteristics perfectly. Other algorithms using the IM framework are the infrequent behaviour-filtering Inductive Miner—infrequent (IMf) [28] and the incompleteness-handling Inductive Miner—incompleteness (IMc) [30].

### Cut detection

Cut definitions are given Appendix 1. Here we describe how the cut detection works. Each of the four process tree operators $\times$, $\rightarrow$, $\wedge$, and $\circlearrowleft$ leaves a different characteristic footprint in the directly-follows graph. Figure 5 visualises these characteristics: for exclusive choice, the activities of one subtree will never occur in the same trace as activities of another subtree. Hence, activities of the different subtrees form clusters that are not connected by edges in the directly-follows graph. Thus, the $\times$ cut is computed by taking the connected components of the directly-follows graph.

If two subtrees are sequentially ordered, all activities of the first subtree strictly precede all activists of the second subtree; in the directly-follows graph we expect to see a chain of clusters without edges going back. The procedure to discover a sequence cut is as follows: each activity starts as a singleton set. First, the strongly connected components of the directly-follows graph are computed and merged. By definition, two activities are in a strongly connected component if they are pairwise reachable, and therefore they cannot sequential. Second, pairwise unreachable sets are merged, as if there is no way to reach two nodes in the same trace, they cannot be sequential. Finally, the remaining sets are sorted based on reachability.

The activities of two parallel subtrees can occur in any intertwined order; we expect all possible connections to be present between the child clusters in the directly-follows graph. To detect parallelism, the graph is negated: the negated graph gets no edge between two activities if both directly-follows edges between these activities are present. If either edge is missing, the negated graph will contain an edge between these two activities. In this negated graph, the partition of the parallel cut is the set of connected components.

In a loop, the directly-follows graph must contain a clear set of start and end activities; all connections between clusters must go through these activities. To detect a loop cut, first the connected components of the directly-follows graph are computed, while excluding the start and end activities. Please note that start and end activities by definition belong to the body of the loop. Second, for each component reachability is used to determine whether it is directed from a start activity to an end activity (body part), or directed the other way round (a redo).

**Fig. 6:**
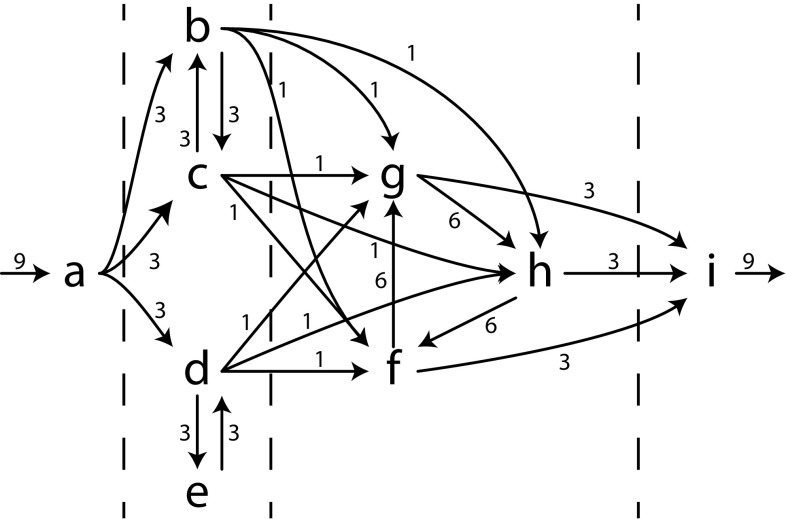
Directly-follows graph $D_1$ of *L*. In a next step, the partition $(\{a\}, \{b, c, d, e\}, \{f, g, h\}, \{i\})$, denoted by the dashed lines, will be used

## Process discovery using a directly-follows graph

Algorithms using the IM framework guarantee soundness, and some even rediscoverability, but do not satisfy the single-pass property, as the log is traversed and even copied during each recursive step. Therefore, we introduce an adapted framework: *Inductive Miner—directly-follows* (IMd framework) that recurses on the directly-follows graph instead of the event log. In this section, we first introduce the IMd framework and a basic algorithm using it. Second, we introduce two more algorithms: one to handle infrequent behaviour and another one that handles incompleteness.

### Inductive Miner: directly-follows

As a first algorithm that uses the framework, we introduce *Inductive Miner—directly-follows* (IMd). We explain the stages of IMd in more detail by means of an example: Let *L* be $[\langle a, b, c, f, g, h, i \rangle$, $\langle a, b, c, g, h, f, i \rangle$, $\langle a, b, c, h, f, g, i \rangle$, $\langle a, c, b, f, g, h, i \rangle$, $\langle a, c, b, g, h, f, i \rangle$, $\langle a, c, b, h, f, g, i \rangle$, $\langle a, d, f, g, h, i \rangle$, $\langle a, d, e, d, g, h, f, i \rangle$, $\langle a, d, e, d, e, d, h, f, g, i \rangle ]$. The directly-follows graph $D_1$ of *L* is shown in Fig. 6.

*Cut detection* IMd searches for a cut that perfectly matches the characteristics mentioned in Sect. 3. As explained, cut detection has been implemented using standard graph algorithms (connected components, strongly connected components), which run in polynomial time, given the number of activities (*O*(*n*)) and directly-follows edges ($O(n^2)$) in the graph.

In our example, the cut $(\rightarrow , \{a\}, \{b, c, d, e\}, \{f, g, h\}, \{i\})$ is selected: as shown in Fig. 5, every edge crosses the cut lines from left to right. Therefore, it perfectly matches the sequence cut characteristic. Using this cut, the sequence is recorded and the directly-follows graph can be split.

**Fig. 7:**
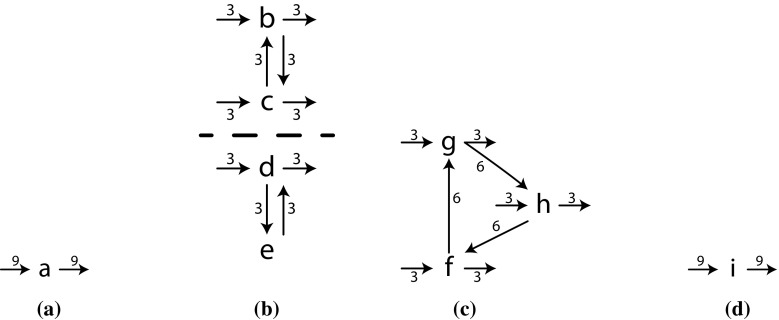
Split directly-follows graphs of $D_1$. The *dashed line* is used in a next step and denotes another partition. (**a**) $D_2$ of $\{a\}$, **b** $D_3$ of $\{b, c, d, e\}$ **c** $D_4$ of $\{f, g, h\}$, **d** $D_5$ of $\{i\}$

*Directly-follows graph splitting*Given a cut, the IMd framework splits the directly-follows graph in disjoint subgraphs. The idea is to keep the internal structure of each of the clusters of the cut by simply projecting a graph on the cluster. Figure 7 shows an example of how $D_1$ (Fig. 6) is split using the sequence cut that was discovered in our example. If the operator of the cut is $\rightarrow$ or $\circlearrowleft$, the start and end activities of a child might be different from the start and end activities of its parent. Therefore, every edge that enters a cluster is counted as a start activity, and an edge leaving a cluster is counted as an end activity. In our example, the start activities of cluster $\{f, g, h\}$ are those having an incoming edge not starting in $\{f, g, h\}$, and correspondingly for end activities. The result is shown in Fig. 7a. In case of $\times$, no edges leave any cluster and hence the start and end activities remain unchanged. In case of $\wedge$, removed edges express the arbitrary interleaving of activities in parallel clusters; removing this interleaving information does not change with which activities a cluster may start or end; thus, start and end activities remain unchanged.

The choices for a sequence cut and the split directly-follows graphs are recorded in an intermediate tree: $\rightarrow ((D_2), (D_3), (D_4), (D_5))$, denoting a sequence operator with 4 unknown subtrees that are to be derived from 4 directly-follows graphs.

*Recursion* Next, IMd recurses on each of the new directly-follows graphs (find cut, split, ...) until a base case (see below) is reached or no perfectly matching cut can be found. Each of these recursions returns a process tree, which in turn can be inserted as a child of an operator identified in an earlier recursion step.

*Base case* Directly-follows graphs $D_2$ (Fig. 7a) and $D_5$ (Fig. 7d) contain base cases: in both graphs, only a single activity is left. The algorithm turns these into leaves of the process tree and inserts them at the respective spot of the parent operator. In our example, detecting the base cases of $D_2$ and $D_5$ yields the intermediate tree $\rightarrow (a, (D_3), (D_4), i)$, in which $D_3$ and $D_4$ indicate directly-follows graphs that are not base cases and will be recursed on later.

*Fall-through* Consider $D_4$ as shown in Fig. 7c. $D_4$ does not contain unconnected parts, so does not contain an exclusive choice cut. There is no sequence cut possible, as *f*, *g*, and *h* form a strongly connected component. There is no parallel cut as there are no dually connected parts and no loop cut as all activities are start and end activities. Thus, IMd selects a fall-through, being a process tree that allows for any behaviour consisting of *f*, *g*, and *h* (a flower model $\circlearrowleft (\tau , f, g, h)$, having the language $(f|g|h)*$). The intermediate tree of our example up till now becomes $\rightarrow (a, (D_3), \circlearrowleft (\tau , f, g, h), i)$ (remember that $\tau$ denotes the activity of which the execution is invisible).

**Fig. 8:**
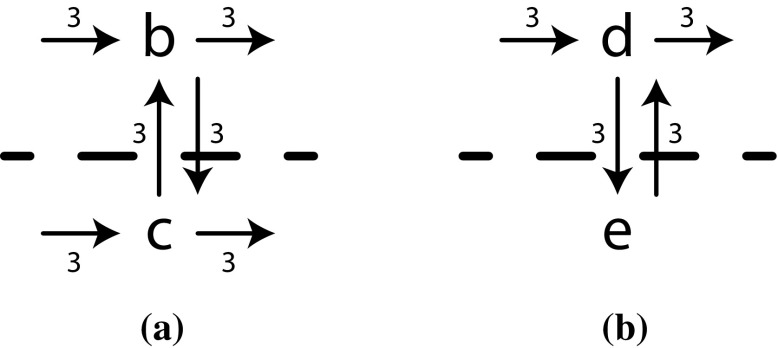
Split directly-follows graphs. Dashed lines denote cuts, which are used in the next steps. **a** $D_6$ of $\{b, c\}$ in $D_3$. **b** $D_7$ of $\{d, e\}$ in $D_3$

*Example continued* In $D_3$, as shown in Fig. 7b, a cut is present: $(\times , \{b, c\}, \{d, e\})$: no edge in $D_3$ crosses this cut. The directly-follows graphs $D_6$ and $D_7$, as shown in Fig. 8a, b, result after splitting $D_3$. The tree of our example up till now becomes $\rightarrow (a, \times ((D_6), (D_7)), \circlearrowleft (\tau , f, g, h), i)$.

In $D_6$, as shown in Fig. 8a, a parallel cut is present, as all possible edges cross the cut, i.e. the dashed line, in both ways. The dashed line in $D_7$ (Fig. 8b) denotes a loop cut, as all connections between $\{d\}$ and $\{e\}$ go via the set of start and end activities $\{d\}$. Four more base cases give us the complete process tree $\rightarrow (a, \times (\wedge (b, c), \circlearrowleft (d, e)), \circlearrowleft (\tau , f, g, h), i)$.

To summarise: IMd selects a cut, splits the directly-follows graph, and recurses until a base case is encountered or a fall-through is necessary. As each recursion removes at least one activity from the graph and cut detection is $O(n^2)$, IMd runs in $O(n^3)$, in which *n* is the number of activities in the directly-follows graph.

By the nature of process trees, the returned model is sound. By reasoning similar to IM [29], IMd guarantees rediscoverability on the same class of models (see Appendix 1), i.e. assuming that the model is representable by a process tree without using duplicate activities, and it is not possible to start loops with an activity they can also end with [29]. This makes IMd the first single-pass algorithm to offer these guarantees.

### Handling infrequency and incompleteness

The basic algorithm IMd guarantees rediscoverability, but, as will be shown in this section, is sensitive to both infrequent and incomplete behaviour. To solve this, we introduce two more algorithms using the IMd framework.

*Infrequent behaviour* Infrequent behaviour in an event log is behaviour that occurs less frequent than ‘normal’ behaviour, i.e. the exceptional cases. For instance, most complaints sent to an airline are handled according to a model, but a few complaints are so complicated that they require ad hoc solutions. This behaviour could be of interest or not, which depends on the goal of the analysis.

Consider again directly-follows graph $D_3$, as shown in Fig. 7b, and suppose that there is a single directly-follows edge added, from *c* to *d*. Then, $(\times , \{b, c\}, \{d, e\})$ is not a perfectly matching cut, as with the addition of this edge the two parts $\{b, c\}$ and $\{d, e\}$ became connected. Nevertheless, as 9 traces showed exclusive choice behaviour and only one did not, this single trace is probably an outlier and in most cases, a model ignoring this trace would be preferable.

To handle these infrequent cases, we apply a strategy similar to IMf [28] and use the IMd framework to define another discovery algorithm: *Inductive Miner—infrequent—directly-follows* (IMfD). Infrequent behaviour introduces edges in the directly-follows graph that violate cut requirements. As a result, a single edge makes it impossible to detect an otherwise very strong cut. To handle this, IMfD first searches for existing cuts as described in Sect. 3.2. If none is found (when IMd would select a fall-through), the graph is filtered by removing edges which are infrequent with respect to their neighbours. Technically, for a parameter $0 \le h \le 1$, for an activity *a* we keep the outgoing edges that occur more than *h* times the most occurring outgoing edge of *a* (a formal definition is given in Appendix 1). Start and end activities are filtered similarly.

**Fig. 9:**
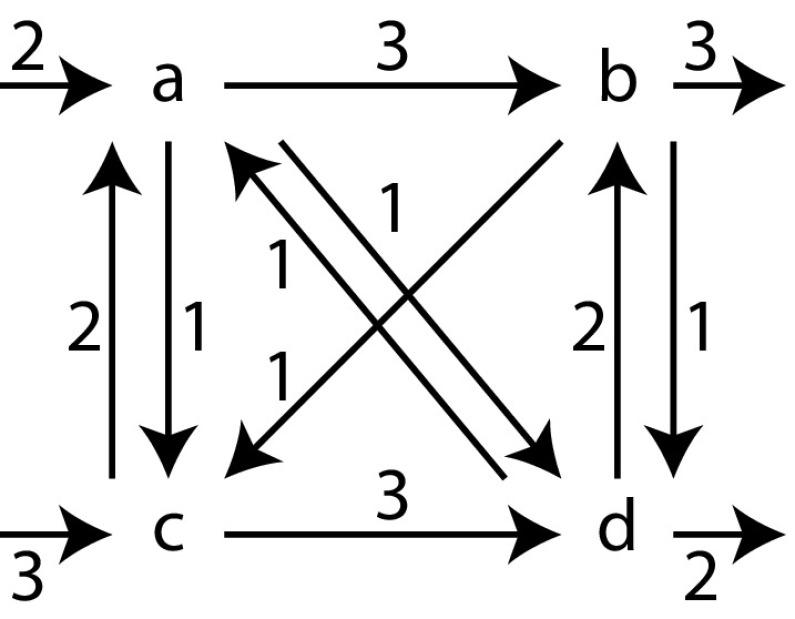
An incomplete directly-follows graph

*Incompleteness* A log in a ‘big-data setting’ can be assumed to contain lots of behaviour. However, we only see example behaviour and we cannot assume to have seen all possible traces, even if we use the rather weak notion of directly-follows completeness [30] as we do here. Moreover, sometimes smaller subsets of the log are considered, for instance when performing slicing and dicing in the context of process cubes [48]. For instance, an airline might be interested in comparing the complaint-handling process for several groups of customers, to gain insight in how the process relates to age, city, and frequent-flyer level of the customer. Then, there might be combinations of age, city, and frequent-flyer level that rarely occur and the log for these customers might contain too little information.

If the log contains little information, edges might be missing from the directly-follows graph and the underlying real process might not be rediscovered. Figure 9 shows an example: the cut $(\{a, b\}, \{c, d\})$ is not a parallel cut as the edge (*c*, *b*) is missing. As the event log only provides example behaviour, it could be that this edge is possible in the process, but has not been seen yet. Given this directly-follows graph, IMd can only give up and return a fall-through flower model, which yields a very imprecise model. However, choosing the parallel cut $(\{a, b\}, \{c, d\})$ would obviously be a better choice here, providing a better precision.

To handle incompleteness, we introduce *Inductive Miner—incompleteness—directly-follows* (IMcD), which adopts ideas of IMc [30] into the IMd framework. IMcD first applies the cut detection of IMd and searches for a cut that perfectly matches a characteristic. If that fails, instead of a perfectly matching cut, IMcD searches for the most probable cut of the directly-follows graph at hand.

IMcD does so by first estimating the most probable behavioural relation between any two activities in the directly-follows graph. In Fig. 9, the activities *a* and *b* are most likely in a sequential relation as there is an edge from *a* to *b*. *a* and *c* are most likely in parallel as there are edges in both directions. Loops and choices have similar local characteristics. For each pair of activities *x* and *y* the probability $P_r(x, y)$ that *x* and *y* are in relation *R* is determined. The best cut is then a partition into sets of activities *X* and *Y* such that the average probabilities that $x \in X$ and $y \in Y$ are in relation *R* is maximal. For a formal definition, please refer to [30].

In our example, the probability of cut $(\wedge , \{a, b\}, \{c, d\})$ is the average probability that (*a*, *c*), (*a*, *d*), (*b*, *c*) and (*b*, *d*) are parallel. IMcD chooses the cut with highest probability, using optimisation techniques. This approach gives IMcD a run-time exponential in the number of activities, but still requires a single pass over the event log.

### Limitations

The IMd framework imposes some limitations on process discovery. We discuss limiting factors on the challenges identified in Sect. 2: rediscoverability, handling incompleteness, handling noise and handling infrequent behaviour, and balancing fitness, precision, generalisation and simplicity.

Limitations on rediscoverability of the IMd framework are similar to the IM framework: the system must be a process tree and adhere to some restrictions, and the log must be directly-follows complete (as discussed before). If the system does not adhere to the restrictions, then IMd framework will not give up but rather try to discover as much process tree like behaviour as possible. For instance, if a part of the process is sequential, then IMd framework might be able to discover this, even though the other parts of the process are not block structured. Therefore, in practice, such models can be useful [8, 15]. To formally investigate what happens on non-block-structured models would be an interesting subject of further study. For the remaining non-block structured parts, the flexibility of IMd framework easily allows for future customisations, e.g. [36].

**Fig. 10:**
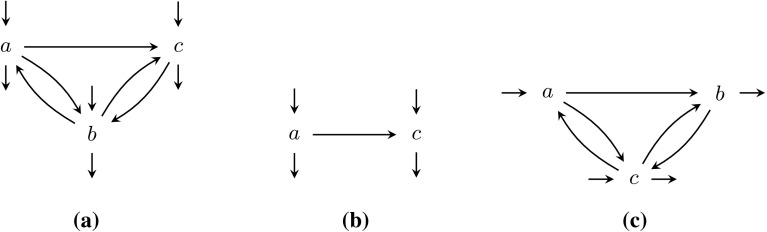
A directly-follows graph that does not suffice to discover concurrency where the log does (**a**, **b**) and an ambiguous directly-follows graph of both $P_{2}$ and $L_{2}$ (**c**). **a** Directly-follows graph of *L*, **b** after splitting by IMd, **c** the directly-follows graph of both $P_2$ and $L_2$

If the log is incomplete, in some cases log-based discovery techniques might handle this incompleteness better. For instance, take the process tree $P_1 = \wedge (a, b, c)$ and an event log $L = \{\langle a, b, c\rangle , \langle c, b, a \rangle , \langle b, a, c\rangle , \langle a, c, b\rangle \}$. Figure 10a shows the directly-follows graph of *L*. Log *L* is not directly-follows complete with respect to $P_1$, as the edge (*c*, *a*) is missing. Both IM and IMd will first detect a concurrent cut $(\wedge , \{a, c\}, \{b\})$. The sublogs after splitting by IM are $\{\langle a, c\rangle , \langle c, a \rangle \}$ and $\{\langle b \rangle \}$, in which the missing directly-follows edge *a*, *c* pops up and enables the rediscovery of $P_1$. In IMd, however, the directly-follows graph is split, resulting in the directly-follows graph for *a*, *c* shown in Fig. 10b, from which $P_1$ cannot be rediscovered. In Sect. 6, we will illustrate that the effect of this limitation is limited on larger examples.

If the log contains noise and/or infrequent behaviour, then the IMd framework might choose a wrong cut at some point (as discussed in Sect. 4.2), possibly preventing rediscovery of the system. The noise handling abilities of log-based and directly-follows-based algorithms differ in detail; in both, noise and infrequent behaviour manifest as superfluous edges in a directly-follows graph. On one hand, in IM, such wrong edges might pop up during recursion by reasoning similar to the incompleteness case (which could be harmful), while using the same reasoning, log-based algorithms might have more information available to filter such edges again (which could be beneficial). In the evaluation, we will investigate this difference further.

Given an event log, both types of algorithms have to balance fitness, precision, generalisation and simplicity. For directly-follows-based algorithms, this balance might be different.

For instance, a desirable property of discovery algorithms is the ability to preserve fitness, i.e. to discover a model that is guaranteed to include all behaviour seen in the event log. For directly-follows-based algorithms, this is challenging. For instance, Fig. 10c shows a complete directly-follows graph of the process tree $P_2 = \wedge (\rightarrow (a, b), c)$. However, it is also the directly-follows graph of the event log $L_2 = \{\langle a, c, b, c, a, b\rangle , \langle c \rangle \}$. Hence, if a fitness-preserving directly-follows-based discovery algorithm would be applied to the directly-follows graph in Fig. 10c, this algorithm could not return $P_2$ and has to seriously underfit/generalise to preserve fitness since the behaviour of both needs to be included. Hence, $P_2$ could never be returned. Therefore, we chose the IMd framework to not guarantee fitness, while the IM framework by its log splitting indirectly takes such concurrency dependencies into account. Please note that this holds for any pure directly-follows-based process discovery algorithm (see the limitations of the $\alpha$-algorithm). Generalisation, i.e. the likelihood that future behaviour will be representable by the model, is similarly influenced.

Algorithms of the IM framework can achieve a high log-precision if it can avoid fall-throughs such as the flower model [28]. Thus, IM framework achieves the highest log-precision if it can find a cut. The same holds for IMd framework, and therefore we expect log-precision to largely depend on the cut selection. In the evaluation, we will investigate log-precision further.

The influence of directly-follows-based algorithms on simplicity highly depends on the chosen simplicity measure: both IM framework and IMd framework return models in which each activity appears once.

## Comparing models to logs and models

We want to evaluate and compare our algorithm to other algorithms regarding several criteria.

- First, we want to compare algorithms based on the size of event logs they can handle, as well as the quality of the produced models. In particular, both recall/fitness and precision (with respect to the given system model or log) need to be compared, as trivial models exist that achieve either perfect recall or perfect precision, but not both.
- Second, we want to assess under which conditions the new algorithms achieve rediscoverability, i.e. under which conditions the partial or incorrect information in the event log allows to obtain a model that has exactly the same behaviour as the original system that produced the event log. More formally, under which conditions (and up to which sizes of systems) has the discovered model the same language as the original system.

Measuring model quality is crucial in typical process mining workflows as shown in the ‘Introduction’. However, as discussed in Sect. 2, existing techniques for measuring model quality (on log or model) cannot handle large and complex logs and processes. Our technique has to overcome two difficulties: (1) it has to compute precision and recall of very large, possibly infinite languages; and (2) it should allow a fine-grained measurement of precision and recall allowing to identify particular activities or behavioural relations where the the model and the log/other model differ.

We first introduce this technique for measuring recall and precision of two models—with the aim of analysing rediscoverability of process mining algorithms (Sect. 5.1). Second, we adopt this technique to also compare a discovered model to a (possibly very large) event log (Sect. 5.2). We use the techniques in our evaluation in Sect. 6.

### Model–model comparison

The *recall* of a model *S* and a model *M* describes the part of the behaviour of *S* that is captured by *M* (compare to the conventional fitness notion in process mining), while *precision* captures the part of the behaviour of *M* that is also possible in *S*.

For a complex model *S* and a complex model *M*, the direct language-based comparison of the two models by constructing and comparing their state spaces might suffer from the state explosion problem, and hence be prohibitively expensive. Therefore, the framework approximates recall and precision by measuring them on subsets of activities, i.e. we avoid the state explosion problem by considering small submodels, and averaging over all such subsets. The framework is applicable to any process model formalism and process discovery algorithm, as long as the languages of the models used can be described as deterministic finite automata (DFAs). We first introduce the general idea of the framework, after which we describe its steps in more detail.

**Fig. 11:**
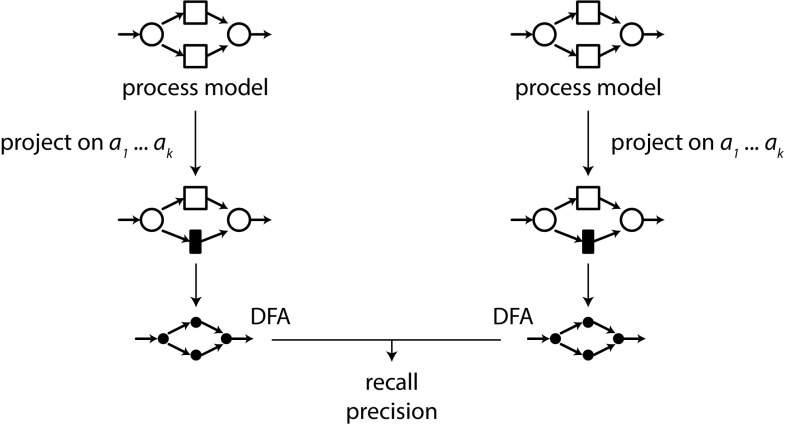
Evaluation framework for process discovery algorithms

*Framework* Figure 11 shows an overview of the model–model evaluation framework; formally, the framework takes as input a model *S*, a model *M*, and an integer *k*. *S* and *M* must be process models, but can be represented using any formalism with executable semantics.

To measure recall and precision of *S* and *M*, we introduce a parameterised technique in which *k* defines the size of the subsets of activities for which recall and precision shall be computed. Take a subset of activities $A = \{a_1\ldots a_k\}$, such that $A \subseteq \varSigma (M) \cup \varSigma (S)$, and $|A| = k$. Then, *S* and *M* are projected onto *A*, yielding $S|_A$ and $M|_A$ (we will show how the projection is performed below). From these projected $S|_A$ and $M|_A$, deterministic finite automata (DFAs) are generated, which are compared to quantify recall and precision. These steps are repeated for all such subsets *A*, and the average recall and precision over all subsets is reported.

As we aim to apply this method to test rediscoverability, a desirable property is that precision and recall should be 1 if and only if $\mathcal {L}(S) = \mathcal {L}(M)$. Theorem 1, given later, states that this is the case for the class of process trees used in this paper.

**Fig. 12:**
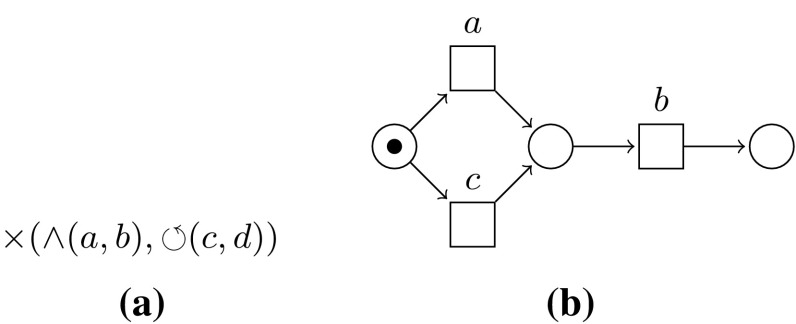
Example models *S* and *M*. **a** Process tree *S*, **b** Petri net *M*

As a running example, we will compare two models, being a process tree and a Petri net. Both are shown in Fig. 12.

In the remainder of this section, we describe the steps of the framework in more detail after which we give an example and prove Theorem 1.

*Projection* Many process formalisms allow for projection on subsets of activities; we give a definition for process trees here and sketch projection for Petri nets in Appendix 5.

A process tree can be projected on a set of activities $A = \{a_1 \ldots a_k\}$ by replacing every leaf that is not in *A* with $\tau$: (in which $\oplus$ is any process tree operator)

$$\begin{aligned} a |_{A}&= \text {if } a \in A \text { then } a \text { else } \tau \\ \tau |_{A}&= \tau \\ \oplus (M_1 \ldots M_n)|_{A}&= \oplus (M_1|_{A} \ldots M_n|_{A}) \end{aligned}$$

After projection, a major problem reduction (and speedup) can be achieved by applying structural language-preserving reduction rules to the process tree, such as the rules described in Appendix 2.

In principle, any further language-preserving state-space reduction rules can be applied; we will not explore further options in this paper.

**Fig. 13:**
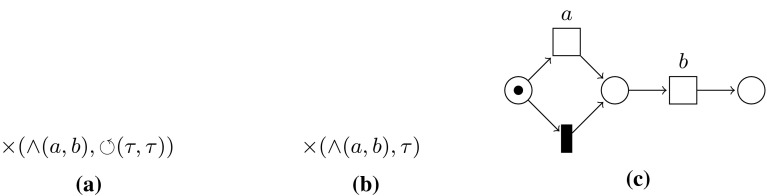
Example models *S* and *M* projected/reduced. **a** $S|_{a, b}$, **b** $S|_{a, b}$ reduced, **c** $M|_{a, b}$

If we project our example process tree and Petri net onto activities *a* and *b*, we obtain the models as shown in Fig. 13.

**Fig. 14:**
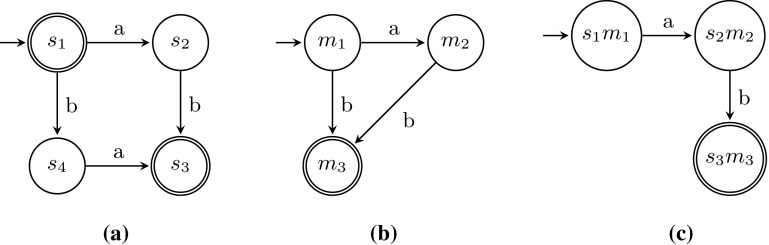
DFAs for *S* and *M* projected to $\{a, b\}$ and reduced, and their conjunction. **a** $\text {DFA}(S|_{\{a, b\}})$, **b** $\text {DFA}(M|_{\{a, b\}})$, **c** $\text {DFAc}(S, M, \{a, b\})$

*Process model to deterministic finite automaton* An *automaton* describes a language based on an alphabet $\varSigma$. The automaton starts in its initial state; from each state, transitions labelled with activities from $\varSigma$ denote the possible steps that can be taken from that state. A state can be an accepting state, which denotes that a trace which execution ends in that state is accepted by the automaton. An automaton with a finite set of states is a *non-deterministic finite automaton* (NFA). In case that the automaton does not contain a state from which two transitions with the same activity leave, the automaton is a *deterministic finite automaton* (DFA). Each NFA can be translated into a DFA and a language for which a DFA exist is a *regular* language; for each DFA, there exists a reduced unique minimal version [35].

Process tree is defined using regular expressions in Appendix 1, which can be transformed straightforwardly into an NFA (we used the implementation [37], which provides a shuffle operator). Second, a simple procedure transforms the NFA into a DFA [35].

The translation of our example $S|_{\{a, b\}}$ and $M|_{\{a, b\}}$ to DFAs results in the automata shown in Fig. 14a, b.

*Comparing deterministic finite automata* Precision is defined as the part of behaviour in $M|_A$ that is also in $S|_A$. Therefore, first the conjunction $\text {DFA}(S|_A) \cap \text {DFA}(M|_A)$ (which we abbreviate to $\text {DFAc}(S, M, A)$) of these DFAs is constructed, which accepts the traces accepted by both $S|_A$ and $M|_A$. Figure 14c shows the conjunctive DFA of our running example. Then, precision is measured similarly to several existing precision metrics, such as [3]: we count the outgoing edges of all states in $\text {DFA}(M|_A)$ and compare that to the outgoing edges of the corresponding states (*s*, *m*) in $\text {DFAc}(S, M, A)$ (for ease of notation, we consider an automaton as a set of states here):

$$\begin{aligned} {\textit{precision}}(S, M, A) =\frac{ \sum _{m \in \text {DFA}(M|_A)} \sum _{(s, m) \in \text {DFAc}(S, M, A)} \text {outgoing edges of } (s, m) \text { in } \text {DFAc}(S, M, A) }{ \sum _{m \in \text {DFA}(M|_A)} \sum _{(s, m) \in \text {DFAc}(S, M, A)} \text {outgoing edges of } m \text { in } \text {DFA}(M|_A) } \end{aligned}$$

If $\text {DFA}(M|_A)$ has no edges at all (i.e. describes the empty language), we define

$$\begin{aligned}&{\textit{precision}}(S, M, A)\\&\quad = {\left\{ \begin{array}{ll} 0 &{} \text {if } \quad \text {DFAc}(S, M, A) \text { has edges }\\ 1 &{} \text {if }\quad \text {DFAc}(S, M, A) \text { has no edges } \end{array}\right. } \end{aligned}$$

Please note that we count acceptance as an outgoing edge, and that states may be counted multiple times if they are used multiple times in $\text {DFAc}$. Recall is defined as the part of behaviour in $S|_A$ that is not in $M|_A$, i.e. $recall(S, M, A) = {\textit{precision}}(M, S, A)$. In our example (see Table 1), recall for (*a*, *b*) is $\frac{1+1+1}{3+1+1+1} = 0.5$; precision is $\frac{1+1+1}{2+1+1} = 0.75$.

**Table 1:** Outgoing edge counting of our running example

Recall for activity subset $\{a, b\}$			
State in $\text {DFA}(S|_{\{a, b\}})$	Outgoing edges	State in $\text {DFAc}(S, M, \{a, b\})$	Outgoing edges
$s_1$	3	$s_1m_1$	1
$s_2$	1	$s_2m_2$	1
$s_3$	1	$s_3m_3$	1
$s_4$	1	–	0

Precision for activity subset $\{a, b\}$			
State in $\text {DFA}(M|_{\{a, b\}})$	Outgoing edges	State in $\text {DFAc}(S, M, \{a, b\})$	Outgoing edges
$m_1$	2	$s_1m_1$	1
$m_2$	1	$s_2m_2$	1
$m_3$	1	$s_3m_3$	1

#### Over all activities

For an alphabet $\varSigma$, finally the previous steps are repeated for each set of activities $\{a_1 \ldots a_k\} \subseteq \varSigma$ of size *k* and the results are averaged:

$$\begin{aligned}&{\textit{recall}}(S, M, k) = \frac{ \sum _{A \subseteq \varSigma (S) \cup \varSigma (M) \wedge |A| = k} {\textit{recall}}(S, M, a_1 \ldots a_k) }{ |\{A \subseteq \varSigma (S) \cup \varSigma (M) \wedge |A| = k\}| }\\&{\textit{precision}}(S, M, k) = {\textit{recall}}(M, S, k) \end{aligned}$$

Note that we assume a closed world here, i.e. the alphabet $\varSigma$ is assumed to be the same for *S* and *M*. If an activity is missing from *M*, we therefore consider *M* to express that the activity can never happen.

*Framework guarantees* Using these definitions, we prove that the framework is able to detect language equivalence between process trees of the class that can be rediscovered by IM and IMd. This theorem will be useful in later evaluations, where from recall and precision being 1, we can conclude that the system was rediscovered.

##### Theorem 1

Let *S* and *M* be process trees without duplicate activities and without $\tau$s. Then, ${\textit{recall}}(S, M, 2) = 1 \wedge {\textit{precision}}(S, M, 2) = 1 \Leftrightarrow \mathcal {L}(S) = \mathcal {L}(M)$.

The proof strategy is to prove the two directions of the implication separately, using that for such trees, there exists a language-unique normal form [29, Corollary 15]. For a detailed proof, see Appendix 3. As for sound free-choice unlabelled workflow nets without short loops the directly-follows graph defines a unique language [61], Theorem 1 applies to these nets as well.

##### Corollary 2

Let *S* and *M* be sound free-choice unlabelled workflow nets without short loops. Then, ${\textit{recall}}(S, M, 2) = 1 \wedge {\textit{precision}}(S, M, 2) = 1 \Leftrightarrow \mathcal {L}(S) = \mathcal {L}(M)$.

Unfortunately, this theorem does not hold for general process trees. For instance, take $S = \times (a, b, c, \tau )$ and $M = \times (a, b, c)$. For $k = 2$, the framework will consider the subtrees $\times (a, b, \tau )$, $\times (a, c, \tau )$ and $\times (b, c, \tau )$ for both *S* and *M*, thus will not spot any difference: ${\textit{recall}} = 1$ and ${\textit{precision}} = 1$, even though the languages of *S* and *M* are clearly different. Only for $k = 3$, the framework will detect the difference.

In Sect. 6, we use the algorithm framework to test incompleteness, noise, and infrequent behaviour on large models. Before that, we first show that the ideas of the framework can also be used to compare models to event logs.

### Log–model comparison

**Fig. 15:**
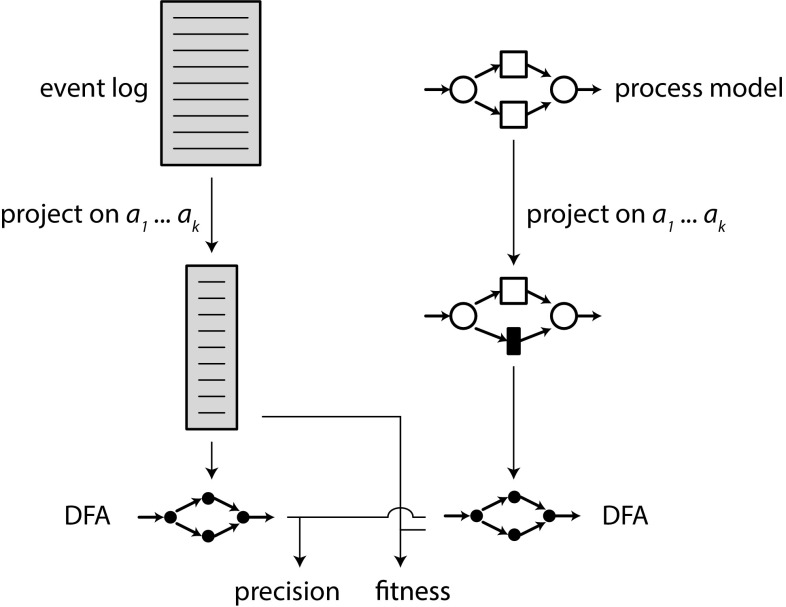
Evaluation approach for logs versus models

In order to evaluate models with respect to event logs, the framework in Sect. 5.1 is adapted as follows: Fig. 15 shows an overview: the framework starts from an event log *L* and a model *M* (in a process mining setting, *M* would have been discovered from *L*). First, *L* and *M* are projected on a set of activities *A*. A log is projected by removing the non-projected events, e.g. $\{\langle a, b, c \rangle , \langle c, d \rangle \}|_{\{a, b\}} = \{\langle a, b \rangle , \langle ~\rangle \}$. Second, from both projections a DFA is constructed. Precision is computed as in Sect. 5.2, i.e. by comparing $\text {DFA}(L|_A)$ and $\text {DFA}(M|_A)$. For fitness, it is desirable that the frequencies of traces are taken into account, such that a trace that appears 10,000 times in *L* contributes more to the fitness value than a trace that appears just once. Therefore, we compute fitness as the fraction of traces of $L|_A$ that can be replayed on $\text {DFA}(M|_A)$:

$$\begin{aligned} {\textit{fitness}}(L, M, A) = \frac{|[ t \in L|_A | t \in \text {DFA}(M|_A) ]|}{|L|_A|} \end{aligned}$$

If the log contains no traces, we define fitness to be 1.

Note that *L* is a multiset: if a trace appears multiple times in *L*, it contributes multiple times as well. This is repeated for all subsets of activities *A* of a certain length *k*, similarly to the model–model comparison. Note that besides a fitness/precision number, the subsets *A* also provide clues where deviations in the log and model occur.

## Evaluation

To understand the impact of ‘big data’ event logs on process discovery and quality assessment, we conducted a series of experiments to answer the following research questions:

1. What is the largest event log (number of events/traces or number of activities) that process discovery algorithms can handle?
2. Are single-pass algorithms such as the algorithms of the IMd framework able to rediscover the system? How large do event logs have to be in order to enable this rediscovery? How do these algorithms compare to classical algorithms?
3. Can the system also be rediscovered if an event log contains unstructured noise or structured infrequent behaviour? How does model quality of the newly introduced algorithms suffer compared to other algorithms?
4. Can pcc framework handle logs that existing measures cannot handle? How do both sets of measures compare on smaller logs?

**Fig. 16:**
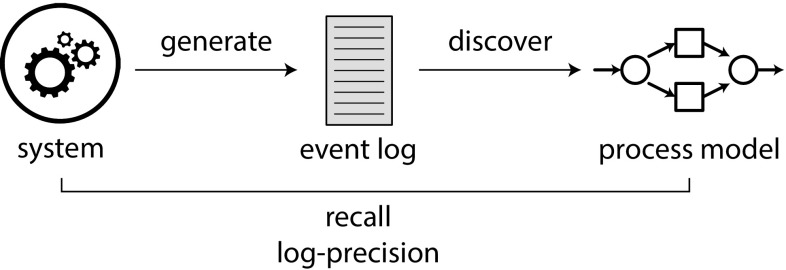
Set-up of our evaluation

To answer the first three questions, we conducted four similar experiments, as shown in Fig. 16: we choose a system, generate an event log, and discover a process model, after which we measure how well the discovered model represents the system using log-precision and recall. Of these experiments, one focuses on scalability, i.e. the ability in handling big event logs and complex systems (Sect. 6.1), one on handling incompleteness (Sect. 6.2), one on handling noise (Sect. 6.3), and one on handling infrequent behaviour (Sect. 6.4).

To answer RQ4, we conducted another experiment: we use real-life logs, apply discovery algorithms, and measure fitness and log-precision, using both the pcc framework and existing measures (Sect. 6.5). All algorithms of the IMd framework and pcc framework are implemented as plug-ins of the ProM framework,^1^ taking as input a directly-follows graph. Directly-follows graphs were generated using an external Python script. For more details on the set-up, please refer to Appendix 4.

### Scalability of IMd versus other discovery algorithms

First, we compare the IMd algorithms with several other discovery algorithms in their ability to handle big event logs and complex systems using limited main memory.

*Set-up* All algorithms were tested on the same set of XES event logs, which have been created randomly from three process trees, of (A) 40 activities, (B) 1000 activities, and (C) 10,000 activities. The three trees have been generated randomly.

For each tree, we first generate a random log of *t* traces, starting *t* at 1. Second, we test whether an algorithm returns a model for that log when allocated 2GB of main memory, i.e. the algorithm terminates with a result and does not crash. If successful, we multiply *t* by 10 and repeat the procedure. The maximum *t* is recorded for each algorithm and process tree A, B, and C.

Besides the new algorithms introduced in this paper, the following algorithms were included in the experiment:

**Table:** 

$\alpha$-Algorithm	($\alpha$)	[49]	ProM 6.5.1a
Heuristics Miner	(HM)	[63]	ProM 6.5.1a
Integer Linear Programming	(ILP)	[54]	ProM 6.5.1a
immediately_follows_cnet_from_log	(P-IF)	[16]	PMLAB
pn_from_ts	(P-PT)	[16]	PMLAB
Inductive Miner	(IM)	[29]	ProM 6.5.1a
Inductive Miner—infrequent	(IMf)	[28]	ProM 6.5.1a
Inductive Miner—incompleteness	(IMc)	[30]	ProM 6.5.1a
IM—directly-follows	(IMd)	This paper	ProM 6.5.1a
IM—infrequent—directly-follows	(IMfD)	This paper	ProM 6.5.1a
IM—incompleteness—directly-follows	(IMcD)	This paper	ProM 6.5.1a

The soundness-guaranteeing algorithms ETM, CCM, and MPM were not included, as ETM is a non-deterministic algorithm and requires long run times to discover reasonable models, and as for CCM and MPM, there is no implementation publicly available. It would be interesting to test these as well.

*Event logs* The complexities of the event logs are shown in Table 2; they were generated randomly from trees A, B, or C. From this table, we can deduce that the average trace length in (A) is 37 events, in (B) 109, and in (C) 764; Appendix 6 shows additional statistics. Thus, the average trace length increases with the number of activities.

The largest log we could generate for A was 217 GB ($10^8$ traces), limited by disk space. For the trees B and C, the largest logs we could generate were $10^6$ and $10^5$ traces, but now limited by RAM. For the bigger logs, the traces were directly transformed into a directly-follows graph and the log itself was not stored. In Table 2, these logs are marked with *.

Compared with [32], tree B was added and the logs of trees A and C were regenerated. Therefore, the log generated for these trees are slightly different from [32]. However, the conclusions were not influenced by this.

**Table 2:** Log complexity ($^{*}$ denotes that a directly-follows graph was generated)

Traces	A: 40 activities		B: 1000 activities complex		C: 10,000 activities more complex	
	Events	Activities	Events	Activities	Events	Activities
1	21	21	190	52	81	66
10	309	40	922	359	8796	1932
$10^2$	3567	40	9577	802	77,664	7195
$10^3$	37,415	40	112,821	973	780,535	9589
$10^4$	370,687	40	1,106,495	999	7,641,398	9991
$10^5$	3,697,424	40	10,908,461	1000	76,663,981	10,000
$10^6$	36,970,718	40	109,147,057	1000	764,585,193	10,000*
$10^7$	369,999,523	40	1,090,802,965	1000*	7,644,466,866	10,000*
$10^8$	3,700,046,394	40	10,908,051,834	1000*	76,477,175,661	10,000*

*Results* Table 3 shows the results. Results that could not be obtained are marked with * (for instance, IMc and IMcD ran for over a week without returning a result).

**Table 3:** Scalability: maximum number of traces an algorithm could handle

	A: 40 activities	B: 1000 activities	C: 10,000 activities
	Traces	Traces	Traces
$\alpha$	10,000	100	1
HM	1,000,000	1,000,000	1
ILP	1000	100	1
P-IF	10,000	0*	1*
P-PT	10,000	0*	1*
IM	100,000	100,000	1000
IMd	100,000,000	100,000,000	100,000,000
IMf	100,000	100,000	1000
IMfD	100,000,000	100,000,000	100,000,000
IMc	100,000$\dagger$	1	10*
IMcD	100,000,000$\dagger$	1	10*

This experiment clearly shows the scalability of the IMd framework, which handles larger and more complex logs easily (IMd and IMfD handle $10^8$ traces, $7\times 10^{10}$ events, and $10^4$ activities). Moreover, it shows the inability of existing approaches to handle larger and complex logs: the most scalable other algorithms were IM and IMf, that both handled only 1000 traces. Furthermore, it shows the limited use sampling would have on such logs (logs manageable for other algorithms, i.e. 1000 traces for tree C do not contain all activities yet). We discuss the results in detail in Appendix 7.

*Time*Timewise, it took a day to obtain a directly-follows graph from the log of $10^8$ traces of tree A, (using the preprocessing Python script) after that discovering a process model was a matter of seconds for IMd and IMfD. For the largest logs they could handle, P-IF, $\alpha$, HM, IM, IMf, and IMc took a few minutes; P-PT took days, ILP a few hours. In comparison, on the logs that ILP could handle, creating a directly-follows graph took a few seconds, just as applying IMd.

### The influence of incompleteness on rediscoverability

To answer RQ2, i.e. whether single-pass algorithms are able to rediscover the system, how large logs need to be in order to enable rediscovery, and how these algorithms compare to classical algorithms, we performed a second experiment. In complex processes, such as B and C, information can be missing from logs if the logs are not large enough: Table 2 shows that in our example, $10^5$ traces were necessary for B to have all activities appear in the log.

**Fig. 17:**
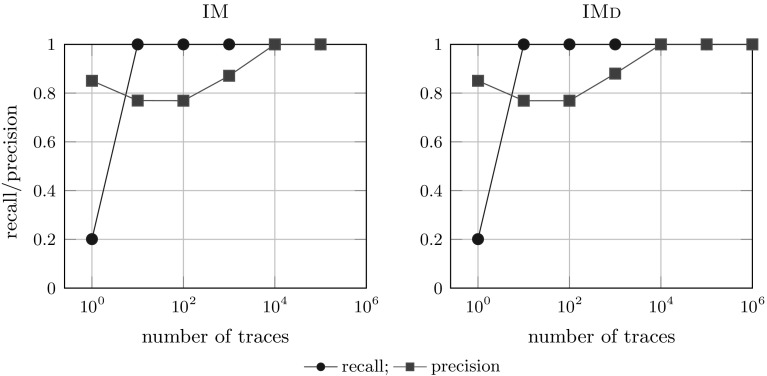
Incompleteness results for process tree A (40 activities)

**Fig. 18:**
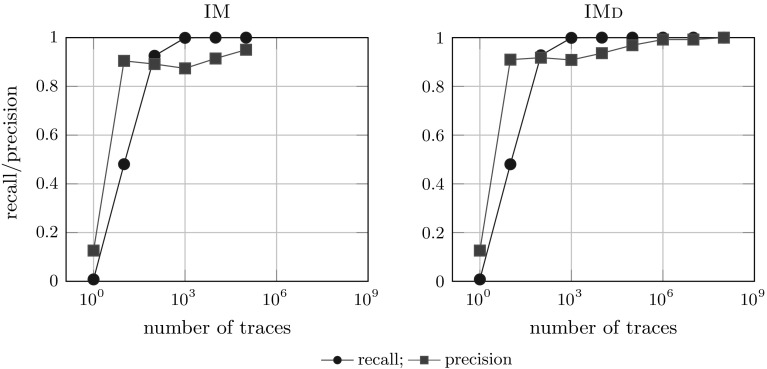
Incompleteness results for process tree B (1000 activities)

*Set-up* For each model generated in the scalability experiment, we measure recall and precision with respect to tree A, B, or C using the pcc framework. Given the results of the scalability experiment, we include the algorithms IM, IMf, IMc, HM, IMd, IMfD, IMcD, and a baseline model allowing for any behaviour (a *flower model*).

As HM does not guarantee to return a sound model, nor provides a final marking, we obtain a final marking using the method described in Appendix 5. However, even with this method we were unable to determine the languages of the models returned by HM, and thus, these were excluded.

*Results* Figure 17 shows that for model A (40 activities) both IM and IMd rediscover the language of A (a model that has 1.0 model-precision and recall with respect to A) on a log of $10^4$ traces. As can be seen in Fig. 18, IMd could rediscover the language of B at $10^8$ traces, IM did not succeed as the largest log it could handle ($10^5$ traces) did not contain enough information to rediscover the language of B. The largest log we generated for tree C, i.e. containing $10^8$ traces, did not contain enough information to rediscover the language of C: IMd discovered a model with a recall of 1.0 and a model-precision of 0.97. Corresponding results have been obtained for IMf/IMfD and IMc/IMcD; see Appendix 8 for all details. The flower model provided the baseline for precision: it achieved recall 1.0 at $10^1$ (A) and $10^2$ (B) traces and achieves a model-precision of 0.8.

We conclude that algorithms of the IMd framework are capable of rediscovering the original system, even in case of very large systems (trees B and C), and that these algorithms do not require larger logs than other algorithms to do so: IM and IMd rediscovered the models on logs of the same sizes; for smaller logs IMd performed slightly better than IMfD. Overall, IMd and IMfD have a similar robustness to incomplete event logs as their IM counterparts, which makes them more robust than other algorithms as well [30]. We discuss the results in detail in Appendix 7.

**Fig. 19:**
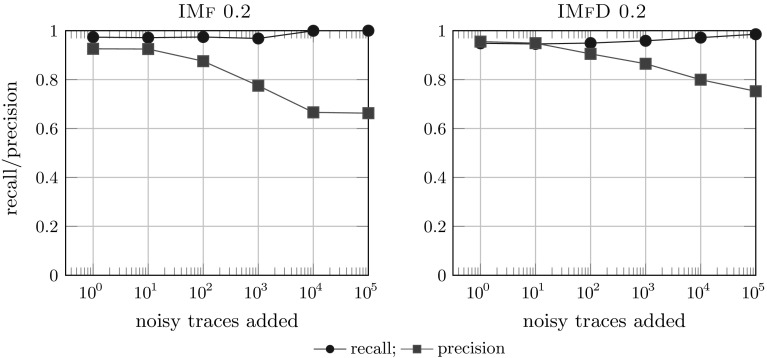
Algorithms applied to logs with noisy traces [tree B (1000 activities)]

### The influence of noise on rediscoverability

To answer RQ3, we tested how noise in the event log influences rediscovery. We took the event log of $10^4$ traces of tree B, as that log was well handled by several algorithms but did not reach perfect recall and precision in the incompleteness experiment, indicating that discovery is possible but challenging. To this $10^4$ traces, we add *n* noisy traces with some noise, for tenfold increasing *n* from 1 to $10^5$, i.e. the logs have 10,001–110,000 traces. A noisy trace is obtained from a normal trace by adding or removing a random event (both with 0.5 probability). By the representational bias of the process trees used in the generation, such a trace is guaranteed to not fit the original model. To each of these logs, we applied IM, IMf, IMd, and IMfD and measured recall and precision with respect to the unchanged system B. No artificial RAM limit was enforced.

Notice that when $10^5$ noisy traces are added, only 9 % of the traces remains noise-free. The directly-follows graph of this noisy log contains 118,262 directly-follows edges, while the graph of the model would just have 32,012. Moreover, almost all activities are observed as start activities (946) and end activities (929) in this log (vs 244/231 in the model). It is clear that without serious noise filtering, no algorithm could make any sense of this log.

*Results* Figure 19 shows the comparison of the 2 noise filtering algorithms IMf and IMfD on the logs of B with various noise levels. Surprisingly, IMfD performs better than IMf: IMfD achieves consistently higher precision at only slight drop in recall compared to IMf whose precision drops to 0.8, which is close to the flower model (i.e. no actual restriction of behaviour). The perfect recall obtained by IM on large lots can be explained by the fall-throughs of IMd and IM: if no cut can be found, a flower model is selected. For IM and IMd, we consistently observed lower precision scores for all models compared to both IMf and IMfD but a consistent fitness of 1.0 (which is easily explained by their lack of noise handling capabilities); exact numbers and more details are available in Appendix 8.

A manual inspection of the models returned shows that all models still give information on the overall structure of the system, while for larger parts of the model no structure could be discovered and a flower submodel was discovered. In this limited experiment, IMfD is the clear winner: it keeps precision highest in return for a little drop in recall. We suspect that this is due to IMfD using less information than IMf and therefore the introduced noise has a larger impact (see Sect. 4.3). More experiments need to evaluate this hypothesis.

### The influence of infrequent behaviour on rediscoverability

To answer the second part of RQ3 we investigated how infrequent behaviour, i.e. structured deviations from the system, influences rediscovery. The set-up of this experiment is similar to the noise experiment, i.e. *t* deviating traces are added. Each deviating trace contains one structural deviation from the model, e.g. for an $\times$, two children are executed. For more details, please refer to Appendix 4.

**Fig. 20:**
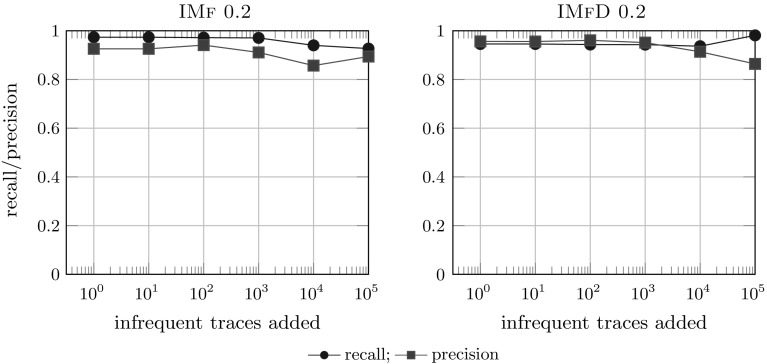
Infrequent behaviour results of process tree B (1000 activities)

Similar to the noise experiment, the log with $10^5$ added infrequent traces has a lot of wrong behaviour: without infrequent behaviour, its directly-follows graph would contain 32,012 edges, 244 start activities, and 231 end activities, but the deviating log contained 118,262 edges, 946 start activities, and 929 end activities. That means that if one would randomly pick an edge from the log, there would be only 27 % chance that the chosen edge would be according to the model. Exact numbers and more details are available in Appendix 8.

*Results* Figure 20 shows the results of IMf and IMfD on logs of process tree B with various levels of added infrequent behaviour. Similar to the noise experiments, IMfD compared to IMf trades recall (0.95 vs 0.99) for log-precision (0.95 vs 0.90). Of the non-filtering versions IM and IMd, both got a recall of 0.99, IM a model-precision of around 0.90, and IMd 0.92, and thus, IMd performs a bit better in this experiment.

We suspect that two of the inserted types of infrequent behaviour positively influenced the results: skipping a child of a $\rightarrow$ or $\wedge$ has no troublesome impact on the directly-follows graph for the IMd framework, but log splitting will introduce a (false) empty trace for the IM framework. The IM framework algorithms must decide to ignore this empty trace in later recursions, while IMd framework algorithms simply don’t see it. Altogether, IMfD performs remarkably well given event logs containing structured deviations from the system.

### Real-life model–log evaluation

To test real-life performance of the new algorithms and to answer RQ4, i.e. whether the newly introduced fitness and precision measures can handle larger logs and how they compare to existing measures, we performed a fourth experiment.

*Experimental set-up* In this experiment, we take four real-life event logs. To these event logs, we apply the algorithms $\alpha$, HM, IM, IMf, IMd, and IMfD and analyse the resulting models manually. The algorithms CCM and MPM are not publicly available and were excluded.

Second, in order to evaluate the pcc framework, we apply the pcc framework and existing fitness [51] and log-precision [1] measures to the discovered models. The models by HM and $\alpha$ were unsound and had to be excluded (we introduced some heuristics for unsound models, but they did not help in this case. For more details, see Appendix 5). Furthermore, IM and IMd do not apply noise filtering and therefore their models are often flower models, so these were excluded as well.

To provide a baseline for log-precision, we add a flower model to the comparison: a flower model allows for all behaviour, so intuitively has the worst precision. Therefore, we scale (normalised) the log-precision according to this baseline:

$$\begin{aligned}&\text {scaled log-precision}\\&\quad = 1-\frac{1-\text {log-precision of model}}{1-\text {log-precision of flower model}} \end{aligned}$$

Intuitively, *scaled precision* denotes the linear precision gain with respect to a flower model, i.e. 0 for the flower model itself and 1 for perfect precision. The pcc framework-fitness measure and the existing fitness measure [51] are conceptually similar, so they are not scaled.

*Logs* We analysed four real-life logs. The first log (BPIC11) originates from the emergency department of a Dutch hospital [56]. BPIC11 describes a fairly unstructured process and contains 624 activities, 1143 traces (patients), and 150,291 events. In our classification, it is a complex and medium log. The second log (BPIC12) originates from a Dutch financial institution and describes a mortgage application process [57]. It contains 23 activities, 13,087 traces (clients), and 164,506 events and is therefore a medium log. BPIC12 was filtered to only contain events having the ‘complete’ life cycle transition, i.e. ‘schedule’ and ‘start’ events were removed. The resulting log was included, as well as the logs for three activity subsets, (BPIC$|_A$, BPIC$|_O$, and BPIC$|_W$), e.g. BPIC$|_A$ only contains activities prefixed by *A*. The third log (SL) originates from the repeated execution of software: SL was obtained by recording method calls executed by RapidMiner, using 5 operators, each corresponding to plug-ins of RapidProM. The recording was performed using the Kieker tool (see http://kieker-monitoring.net) and repeated 25 times with different input event logs. In total, the event log (*SL*) has 25 traces, 5,869,492 events, and 271 activities, which makes it a large. Its traces are particularly long: up to 1,151,788 events per trace. The fourth log (CS) originates from a website of a dot-com start-up and represents click-stream data, i.e. every website visitor is a trace, and each page visited is an events [26]. CS contains 77,513 traces, 358,278 events, and 3300 activities, which makes it more complex and medium. As described in the ‘Introduction’, much bigger event logs exist. We were able to run IMd and IMfD on larger and more complex logs, but we were unable to compute metrics or even visualise the resulting models. Therefore, we do not report on such logs in this evaluation.

**Fig. 21:**
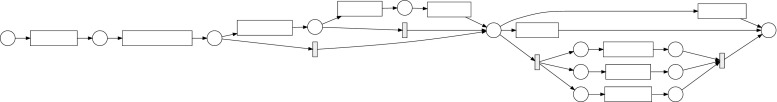
IMf applied to BPIC12$|_A$ (without activity names)

**Fig. 22:**
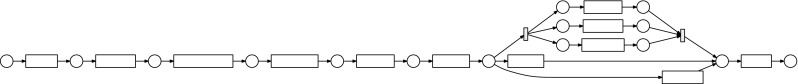
IMfD applied to BPIC12$|_A$ (without activity names)

**Fig. 23:**
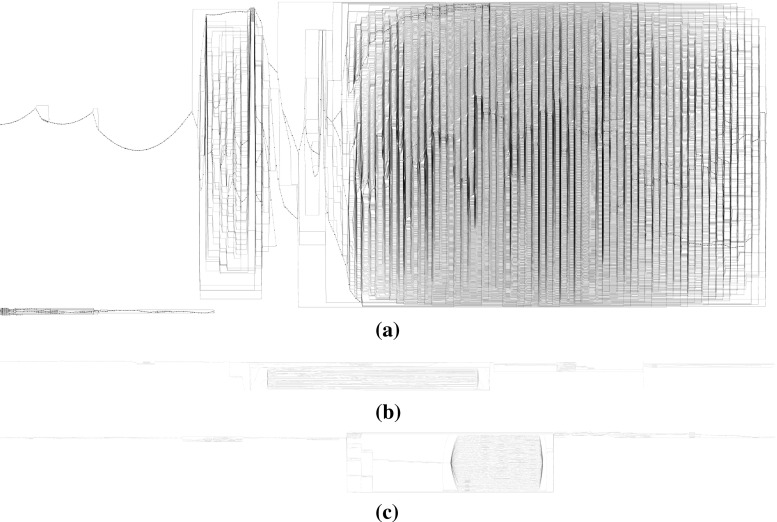
Models discovered from a software execution log. **a** Impression of the model discovered by HM. **b** Impression of the model discovered by IMf. **c** Impression of the model discovered by IMfD.

*Results for process discovery* The first step in this experiment was to apply several process discovery algorithms.

On BPIC11, IM, IMf, IMd, and IMfD produced a model.

**Table 4:** Log measures compared on real-life logs

		Existing techniques				This paper (pcc framework)			
		Fitness [51]	Log-precision [1]		Time	Fitness	Log-precision		Time
			Measured	Scaled			Measured	Scaled	
BPIC11	IMf	Out of memory				0.627	0.764	0.472	25 s
	IMfD	Out of memory				0.997	0.766	0.477	1 m
	Flower	1.000	0.002	0.000	5 h	1.000	0.553	0.000	25 s
BPIC12$|_A$	IMf	0.995	0.606	0.940	$\le$1 s	0.999	0.967	0.931	$\le$1 s
	IMfD	0.816	1.000	1.000	$\le$1 s	0.700	1.000	1.000	$\le$1s
	Flower	1.000	0.227	0.000	$\le$1 s	1.000	0.520	0.000	$\le$1 s
BPIC12$|_O$	IMf	0.991	0.508	0.351	$\le$1 s	0.981	0.809	0.407	$\le$1 s
	IMfD	0.861	0.384	0.187	$\le$1 s	0.862	0.794	0.360	$\le$1 s
	Flower	1.000	0.242	0.000	$\le$1 s	1.000	0.678	0.000	$\le$1 s
BPIC12$|_W$	IMf	0.876	0.690	0.553	$\le$1 s	0.875	0.836	0.611	$\le$1 s
	IMfD	0.914	0.300	$-$0.010	$\le$1 s	0.923	0.823	0.581	$\le$1 s
	Flower	1.000	0.307	0.000	$\le$1 s	1.000	0.578	0.000	$\le$1 s
BPIC12	IMf	0.967	0.364	0.290	20 m	0.978	0.668	0.092	$\le$1 s
	IMfD	1.000	0.189	0.095	25 m	1.000	0.693	0.161	$\le$1 s
	Flower	1.000	0.104	0.000	30 m	1.000	0.634	0.000	$\le$1 s
SL	IMf	Out of memory				0.584	0.246	$-$0.158	30 m
	IMfD	Out of memory				0.924	0.385	0.055	30 m
	Flower	Out of memory				1.000	0.349	0.000	35 m
CS	IMf	Out of memory				0.999	0.580	0.023	1h
	IMfD	Out of memory				0.999	0.585	0.036	6.5 h
	Flower	Out of memory				1.000	0.570	0.000	55 m

On BPIC12, all the algorithms produced a model. We illustrate the results of the experiments on BPIC12, filtered for activities starting with *A* (BPIC$|_A$): Fig. 21 shows the model returned by IMf; Fig. 22 the model by IMfD. This illustrates the different trade-offs made between IM and IMd: these models are very similar, except that for IMf, three activities can be skipped. Operating on the directly-follows abstraction of the log, IMfD was unable to decide to make these activities skippable, which lowers fitness a bit (0.816 vs 0.995) but increases precision (1 vs 0.606).

**Fig. 24:**
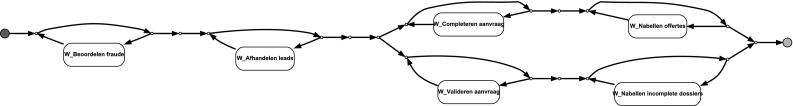
IMfD applied to BPIC$|_W$

On the SL log, HM, IM, IMf, IMd, and IMfD produced a model, and $\alpha$ could not proceed beyond passing over the event log. The models discovered by HM, IMf, and IMfD are shown in Fig. 23 (we uploaded these models to http://www.processmining.org/blogs/pub2015/scalable_process_discovery_and_evaluation). The model discovered by HM has 2 unconnected parts, of which one part cannot be executed. Hence, it is not a workflow model, thus not sound and, as discussed before, difficult to be analysed automatically. In the models discovered IMf and IMfD, the five RapidProM operators are easily recognisable. However, the models are too complex to be analysed in detail by hand.^2^ Therefore, in further analysis steps, problematic parts of the models by IMf and IMfD could be identified, the log filtered for them, and the analysis repeated.

On the CS log, IM, IMf, IMd, and IMfD produced a model. IMd and IMfD returned a model in less than 30 s using less than 1 GB of RAM, while IM and IMf took more than an hour and used 30 GB of RAM. As CS has five times more activities than SL, we could not visualise it. This illustrates that scalable process discovery is a first step in scalable process mining: the models we obtained are suitable for automatic processing, but human analysis without further visualisation techniques is very challenging.

*Results for log conformance checking* Table 4 shows the results, extended with the approximate running time of the techniques.

Fitness scores according to the pcc framework differ from the fitness scores by van der Aalst [51] by at most 0.05 (except for BPIC12$|_A$ IMfD). Thus, this experiment suggests that the new fitness measurement could replace the alignment-based fitness [51] metric, while being generally faster on both smaller and larger logs, though additional experiments may be required to verify this hypothesis. More importantly, the pcc framework could handle logs (BPIC11, SL, CS) that the existing measure could not handle.

Comparing the scaled precision measures, the pcc framework and the existing approach agree on the relative order of IMf and IMfD for BPIC12$|_A$ and BPIC12$|_O$, disagree on BPIC12, and are incomparable on BPIC11, SL, and CS due to failure of the existing measure. For BPIC12$|_W$, IMfD performed *worse* than the flower model according to Adriansyah et al. [1] but *better* according to our measure. This model, as shown in Fig. 24, is certainly more restrictive than a flower model, which is correctly reflected by our new precision measure. Therefore, likely the approach of Adriansyah et al. [1] encounters an inaccuracy when computing the precision score. For BPIC12, precision [1] ranks IMf higher than IMfD, whereas our precision ranks IMfD higher than IMf. Inspecting the models, we found that IMf misses one activity from the log while IMfD has all activities. Apparently, our new measure penalises more for a missing activity, while the alignment-based existing measure penalises more for a missing structure.

A similar effect is visible for SL: IMf achieves a lower precision than the flower model. Further analysis revealed that several activities were missing from the model by IMf. The following example illustrates the effect: let $L = \{\langle a, b \rangle \}$ be a projected log and $M = a$ a projected model. Then, technically, their conjunction is empty and hence both precision and recall are 0. This matches intuition, as they have no trace in common. This sensitivity to missing activities is inherent to language-based measuring techniques. From the model discovered by IMf, 45 activities are missing, which means that of the 36,585 pairs of activities that are considered for precision and recall, in 11,160 pairs a missing activity is involved.

This experiment does not suggest that our new measure can directly replace the existing measures, but precision seems to be able to provide a categorisation, such as good/mediocre/bad precision, compared to the flower model.

Altogether, we showed that our new fitness and precision metrics are useful to quickly assess the quality of a discovered model and decide whether to continue analyses with it or not, in particular on event logs that are too large for current techniques. In addition to simply providing an aggregated fitness and precision value, both existing and our new technique allow for more fine-grained diagnostics of *where* in the model and event log fitness and precision are lost. For instance, by looking at the subsets $a_1 \ldots a_k$ of activities with a low fitness or precision score, one can identify the activities that are not accurately represented by the model and then refine the analysis of the event log accordingly.

For most event logs, IMfD seems to perform comparably to IMf. However, please notice that by the nature of fitness and log-precision, for each event log there exists a trivial model that scores perfectly on both, i.e. the model consisting of a choice between all traces. As such a model provides neither any new information nor insight, generalisation and simplicity have to be taken into account as well. As future work, we would like to adapt generalisation metrics to be applicable to large event logs and complex processes as well.

## Conclusion

Process discovery aims to obtain process models from event logs, while conformance checking aims to obtain information from the differences between a model and either an event log or a system model. Currently, there is no process discovery technique that works on larger and more complex logs, i.e. containing billions of events or thousands of activities, and that guarantees both soundness and rediscoverability. Moreover, current log conformance checking techniques cannot handle medium and complex logs, and as current process discovery evaluation techniques are based on log conformance checking, algorithms cannot be evaluated for medium and complex logs. In this paper, we pushed the boundary on what can be done with larger and more complex logs.

For process discovery, we introduced the *Inductive Miner—directly-follows* (IMd) framework and three algorithms using it. The input of the framework is a directly-follows graph, which can be obtained from any event log in linear time, for instance using highly scalable techniques such as map-reduce. The IM framework uses a divide-and-conquer strategy that recursively builds a process model by splitting the directly-follows graph and recursing on the subgraphs until it encounters a base case.

We showed that the memory usage of algorithms of the IMd framework is independent of the number of traces in the event log considered. In our experiments, the scalability was only limited by the logs we could generate. The IMd framework managed to handle over 70 billion events, while using only 2 GB of RAM; some other techniques required the event log to be in main memory and therefore could handle at most 1–10 million events. Besides scalability, we also investigated how the new algorithms compare qualitatively to existing techniques that use more knowledge, but also have higher memory requirements. The new algorithms handled systems of 10,000 activities in polynomial time and were robust to incompleteness, noise, and infrequent behaviour. Moreover, they always return sound models and suffered little loss in quality compared to multipass algorithms; in some cases we even observed quality improvements.

For conformance checking, we introduced the *projected conformance checking framework* (pcc framework), that is applicable to both log–model and model–model conformance checking. The pcc framework measures recall/fitness and precision, by projecting both system and system model/log onto subsets of activities to determine their recall/fitness and precision. Using this framework, one can measure recall/fitness and precision of arbitrary models with a bounded state space of (almost) arbitrary size.

The pcc framework’s model–model capabilities enable a novel way to evaluate discovery techniques that scales well and provides new insights. We applied this to test robustness of various algorithms to incompleteness, noise, and infrequent behaviour. Moreover, we showed that the log–model version of the pcc framework allows to measure fitness and precision of a model with respect to an event log, even in cases where classical techniques fail, and can give detailed insights into the location of deviations in both log and model.

Altogether, we have presented the first steps of process mining workflows on very large data sets: discovering a model and assessing its quality. However, as we encountered in our evaluation, we envision further steps in the processing and visualisation of large models, such as using natural language-based techniques [5]. To ease the analyses in contexts of big data, our algorithm evaluation framework could be combined with the approach in [45], by having our framework detecting the problematic sets of activities, and the approach in [45] focusing on these submodels. For instance, it would be interesting to approximate performance measures on the model without computing alignments.

Furthermore, it would be interesting to study the influence of *k* on the pcc framework, both practically and theoretically. As shown in Sect. 5.1, there exist cases for which the language equivalence can only be guaranteed if *k* is at least the number of nodes minus one. However, besides the classes for which Theorem 1 or Corollary 2 holds, there might be other classes of models for which a smaller *k* suffices.

## Figures and Tables

**Fig. 25:**
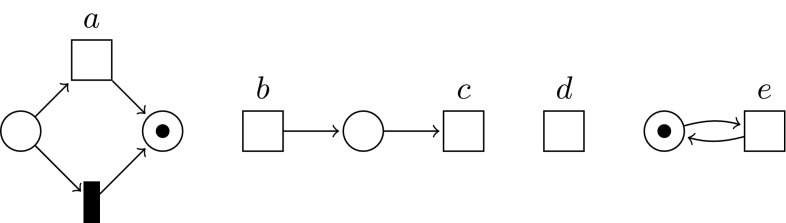
An unbounded Petri net in a conditional deadlock

**Fig. 26:**
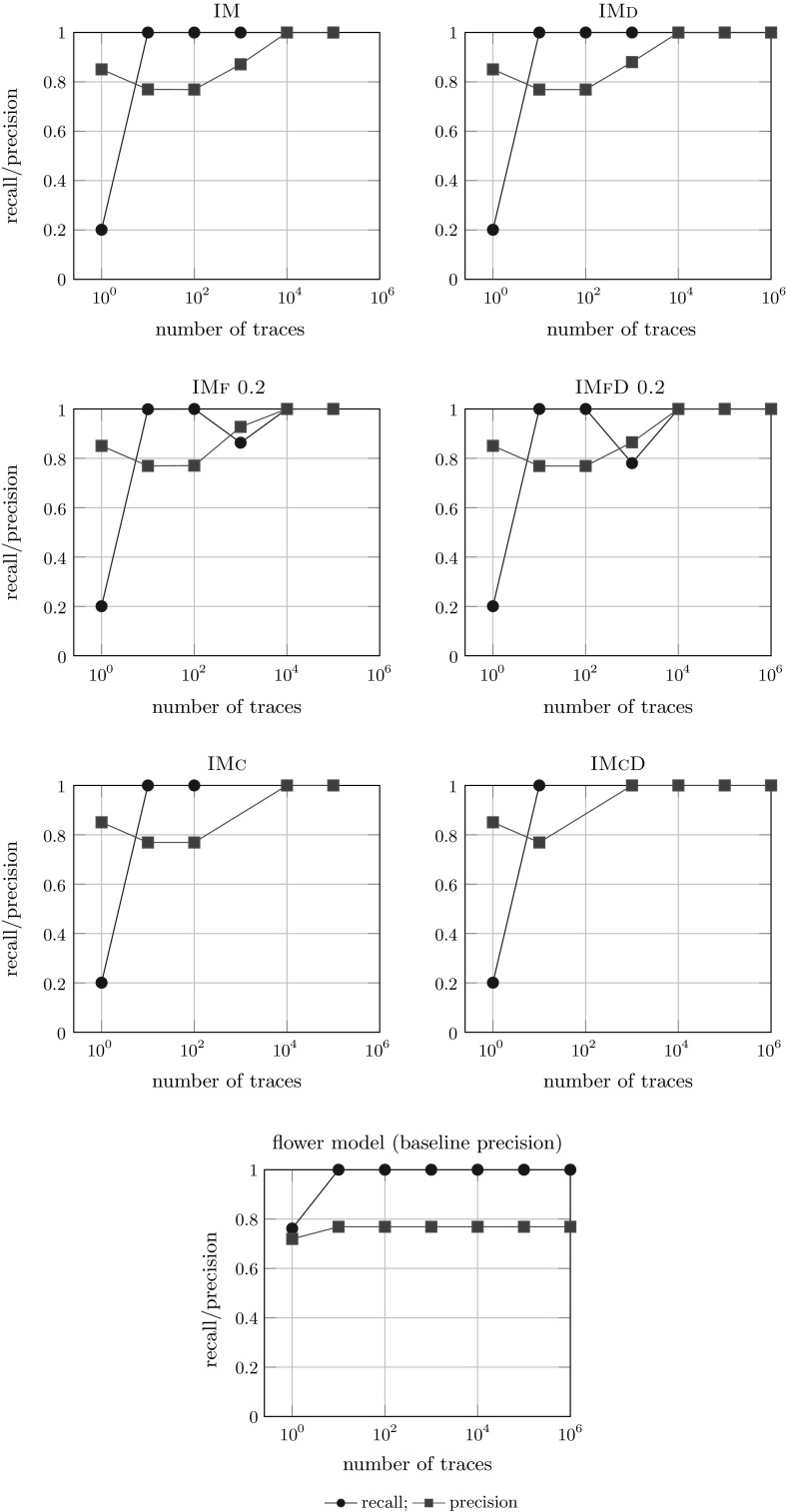
Incompleteness results for process tree A (40 activities)

**Fig. 27:**
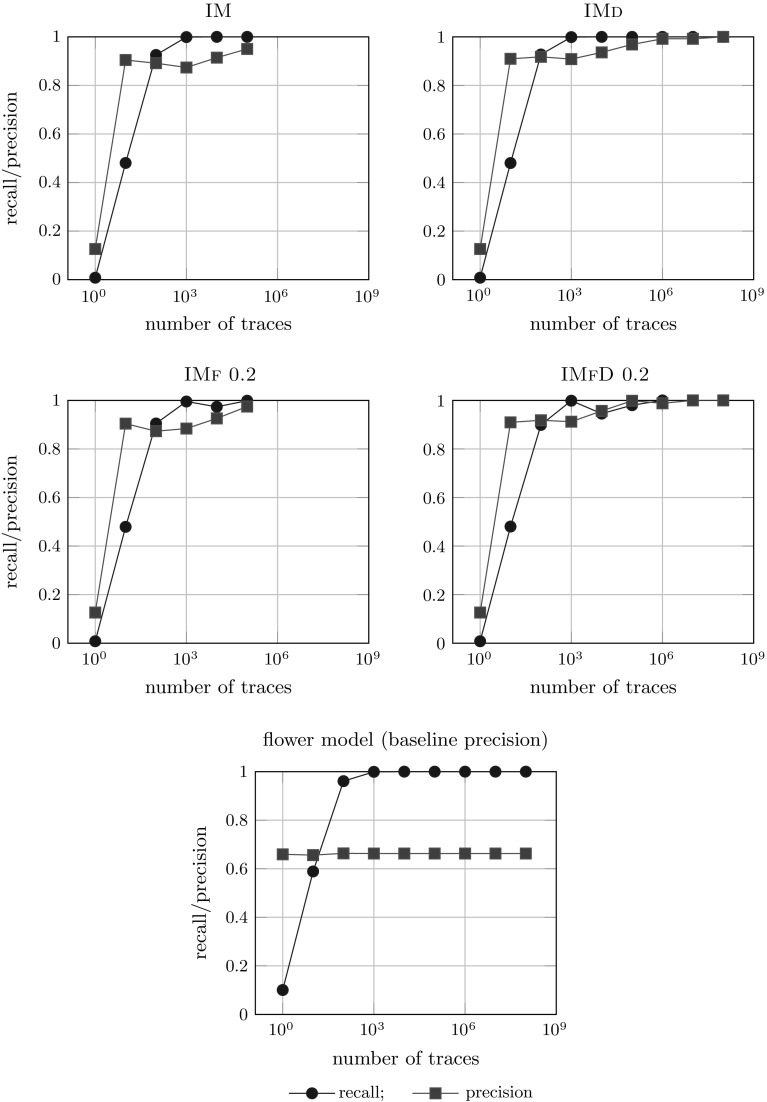
Incompleteness results for process tree B (1000 activities)

**Fig. 28:**
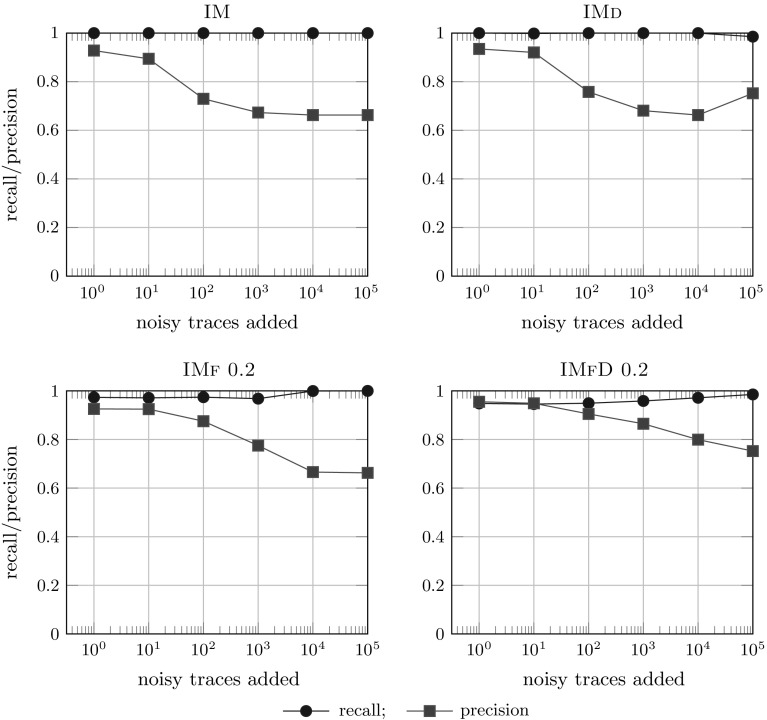
Algorithms applied to logs with noisy traces [tree B (1000 activities)]

**Fig. 29:**
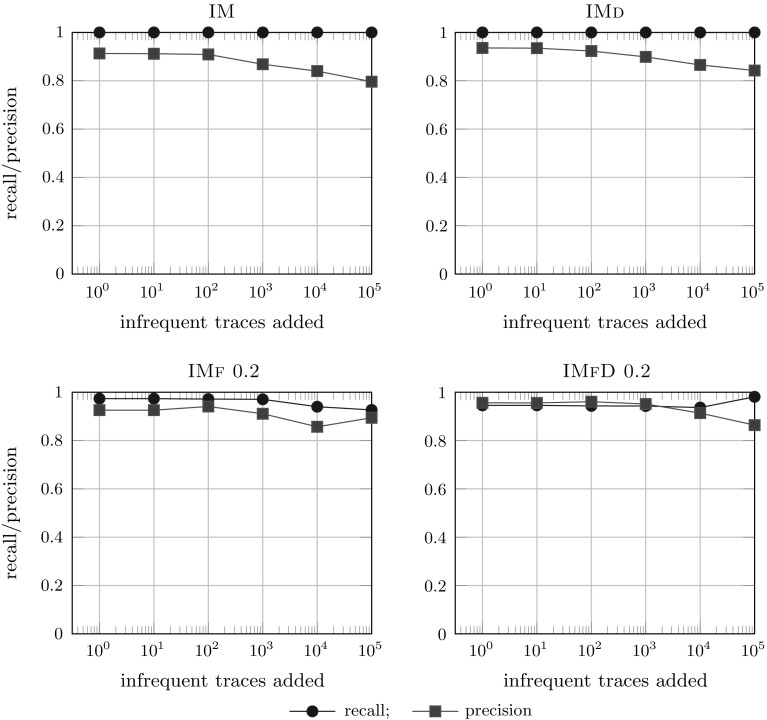
Infrequent behaviour results of process tree B (1000 activities)

## Appendix 1: Formal definitions

### Process trees

Recursively define the shuffle product operator $\thicksim$ on traces as follows:

$$\begin{aligned}&\langle ~\rangle \thicksim B ={} \{B\}\\&A \thicksim \langle ~\rangle ={} \{A\}\\&(\langle a \rangle \cdot A) \thicksim (\langle b \rangle \cdot B)\\&\quad ={} \{\langle a \rangle \} \cdot (A \thicksim (\langle b \rangle \cdot B)) \cup \{\langle b \rangle \} \cdot ((\langle a \rangle \cdot A) \thicksim B) \end{aligned}$$

Then, the shuffle product can also be applied to languages:

$$\begin{aligned} L_1 \thicksim L_2 = \bigcup _{w_1 \in L_1 \wedge w_2 \in L_2} w_1 \thicksim w_2 \end{aligned}$$

Using these, we define the semantics of process trees using their language (taken from [30]):

$$\begin{aligned}&\mathcal {L}(\tau ) = \{ \langle ~\rangle \}\\&\mathcal {L}(a) = \{\langle a \rangle \} \text { for } a \in \varSigma \\&\mathcal {L}(\times (M_1, \ldots , M_n)) = \mathcal {L}(M_1) | \mathcal {L}(M_2) \ldots \mathcal {L}(M_n)\\&\mathcal {L}(\rightarrow (M_1, \ldots , M_n)) = \mathcal {L}(M_1) \cdot \mathcal {L}(M_2) \cdots \mathcal {L}(M_n)\\&\mathcal {L}(\wedge (M_1, \ldots , M_n)) = \mathcal {L}(M_1) \thicksim \mathcal {L}(M_2) \ldots \mathcal {L}(M_n)\\&\mathcal {L}(\circlearrowleft (M_1, \ldots , M_n)) \\&\quad = \mathcal {L}(M_1) \cdot (\mathcal {L}(\times (M_2, \ldots , M_n)) \cdot \mathcal {L}(M_1))^* \end{aligned}$$

### Inductive Miner framework (IM framework)

Let $\mathbb {L}$ be the set of all logs, $\mathbb {P}$ the set of all process trees and $\bigoplus = \{\times , \rightarrow , \wedge , \circlearrowleft \}$. The Inductive Miner framework (IM framework) takes as input a cut selection procedure $findCut: \mathbb {L} \rightarrow \bigoplus \times 2^\varSigma$, a log splitting function $split : \mathbb {L} \times \bigoplus \times 2^\varSigma \rightarrow 2^\mathbb {L}$, a fall-through function $fallThrough: \mathbb {L} \rightarrow \mathbb {P}$, and a base-case generating function: $findBaseCase: \mathbb {L} \rightarrow \mathbb {P}$. The functions *findCut* and *findBaseCase* may fail to produce a result.

Each of the algorithms, i.e. IM, IMf and IMc, provides a specific set of these functions.

### Inductive Miner: directly-follows framework (IMd framework)

Let $\mathbb {G}$ be the set of all directly-follows graphs, $\mathbb {P}$ the set of all process trees and $\bigoplus = \{\times , \rightarrow , \wedge , \circlearrowleft \}$. The Inductive Miner—directly-follows framework (IMd framework) takes as input a cut selection procedure $findCut_d: \mathbb {G} \rightarrow \bigoplus \times 2^\varSigma$, a log splitting function $split_d : \mathbb {G} \times \bigoplus \times 2^\varSigma \rightarrow 2^\mathbb {G}$, a fall-through function $fallThrough_d: \mathbb {G} \rightarrow \mathbb {P}$, and a base-case generating function: $findBaseCase_d: \mathbb {G} \rightarrow \mathbb {P}$. The functions $findCut_d$ and $findBaseCase_d$ may fail to produce a result.

Each of the algorithms, i.e. IMd, IMfD and IMcD, provides a specific set of these functions.

### Class of models that can be rediscovered

The following definitions are taken from [29]. Let *S* be a system. Then, *S* can be rediscovered by IM and IMd if there exists a process tree $S'$ such that $\mathcal {L}(S) = \mathcal {L}(S')$, and (in which $\oplus (S'_1, \ldots , S'_n)$ is a node at any position in $S'$, *Start*(*M*) denotes the start activities of a model *M* and *End*(*M*) the end activities.)

1. Duplicate activities are not allowed: $\forall i \ne j : \varSigma (S'_i) \cap \varSigma (S'_j) = \emptyset$.
2. If $\oplus =\, \circlearrowleft$, the sets of start and end activities of the first branch must be disjoint: $\oplus =\, \circlearrowleft \, \Rightarrow Start(S'_1) \cap End(S'_1) = \emptyset$.
3. No $\tau$’s are allowed: $\forall i \le n : S'_i \ne \tau$.

The rediscoverability proof for this class of models is given in Leemans et al. [29].

### Inductive Miner: directly-follows (IMd)

The following definitions are taken from Leemans et al. [29]. In these definitions, $a \mapsto b$ denotes that there is a directly-follows edge from *a* to *b*; $a \mapsto ^+b$ denotes that there is an edge in the transitive closure. Moreover, *Start* denotes the set of start activities, *End* the set of end activities. The function $findCut_{d, {IMd}{}}(G)$ searches for a cut that perfectly matches the definitions given below, using efficient procedures as described Sect. 3.2.

#### Definition 1

An *exclusive choice cut* is a cut $(\times , \varSigma _1, \ldots , \varSigma _n)$ such that

#### Definition 2

A *sequence cut* is an ordered cut $(\rightarrow , \varSigma _1, \ldots , \varSigma _n)$ such that

2. $$\forall 1 \le i < j \le n, a_i \in \varSigma _i, a_j \in \varSigma _j : a_i \mapsto ^+a_j $$

#### Definition 3

A *concurrent cut* is a cut $(\wedge , \varSigma _1, \ldots , \varSigma _n)$ such that

1. $$\forall i : \varSigma _i \cap Start \ne \emptyset \wedge \varSigma _i \cap End \ne \emptyset $$
2. $$\forall i \ne j, a_i \in \varSigma _i, a_j \in \varSigma _j : a_i \mapsto a_j \wedge a_j \mapsto a_i $$

#### Definition 4

A *loop cut* is a partially ordered cut $(\circlearrowleft , \varSigma _1, \ldots , \varSigma _n)$ such that

1. $$Start \cup End \subseteq \varSigma _1 $$
2. $$\forall i \ne 1, a_i \in \varSigma _i, a_1 \in \varSigma _1 : a_1 \mapsto a_i \Rightarrow a_1 \in End $$
3. $$\forall i \ne 1, a_i \in \varSigma _i, a_1 \in \varSigma _1 : a_i \mapsto a_1 \Rightarrow a_1 \in Start $$
5. $$\forall i \ne 1, a_i \in \varSigma _i, a_1 \in Start(G) : \big ( \exists a_1' \in \varSigma _1 : a_i \mapsto a_1' \big ) \Leftrightarrow a_i \mapsto a_1 $$
6. $$\forall i \ne 1, a_i \in \varSigma _i, a_1 \in End(G) : \left( \exists a_1' \in \varSigma _1 : a_1' \mapsto a_i \right) \Leftrightarrow a_1 \mapsto a_i $$

### Inductive Miner: infrequent—directly-follows (IMfD)

For Inductive Miner—infrequent—directly-follows (IMfD), the cut finding function $findCut_{d, {IMfD}{}}(G)$ first tries the IMd cut finding function. If that fails, it filters the directly-follows graph and tries again. In the following definition, $w(a \mapsto b)$ denotes the weight of edge $a \mapsto b$ in *G*, and *l* denotes a noise threshold value ($0 \le l \le 1)$.

$$\begin{aligned} filter(G, l)&= \{ a \mapsto b | a \mapsto b \in G \wedge w(a \mapsto b) \\&\ge \max _c(w(a \mapsto c)) \cdot l \} \end{aligned}$$

$$\begin{aligned}&findCut_{d, \text {{IMfD}{}}}(G)\\&\quad = \left\{ \begin{array}{l@{\quad }l} findCut_{d, \text {{IMd}{}}}(G) &{} \text {if successful} \\ findCut_{d, \text {{IMd}{}}}(filter(G, l)) &{} \text {otherwise} \\ \end{array} \right. \end{aligned}$$

## Appendix 2: Reduction

In the model evaluation framework, the to-be compared models *S* and *M* are first projected by replacing activities by $\tau$. In order to efficiently compare the languages of these projected models, we apply several language-preserving reduction rules. In addition to the first six language-unique reduction rules introduced in Leemans et al. [29], several new reduction rules are applied (in which $\oplus$ is any process tree operator):

$$\begin{aligned} \oplus (M) ={}&M \\ \times (\ldots _1, \times (\ldots _2), \ldots _3) ={}&\times (\ldots _1, \ldots _2, \ldots _3) \\ \rightarrow (\ldots _1, \rightarrow (\ldots _2), \ldots _3) ={}&\rightarrow (\ldots _1, \ldots _2, \ldots _3) \\ \wedge (\ldots _1, \wedge (\ldots _2), \ldots _3) ={}&\wedge (\ldots _1, \ldots _2, \ldots _3) \\ \circlearrowleft (\circlearrowleft (M, \ldots _1), \ldots _2) ={}&\circlearrowleft (M, \ldots _1, \ldots _2)\\ \circlearrowleft (M, \ldots _1, \times (\ldots _2), \ldots _3) ={}&\circlearrowleft (M, \ldots _1, \ldots _2, \ldots _3) \\ \hline \rightarrow (\ldots _1, \tau , \ldots _2) ={}&\rightarrow (\ldots _1, \ldots _2) \\&\text { if } \ldots _1 \text { or } \ldots _2 \text { non-empty}\\ \wedge (\ldots _1, \tau ) ={}&\wedge (\ldots _1) \\&\text { if } \ldots _1 \text { non-empty}\\ \times (\tau , M, \ldots _1) ={}&\times (M, \ldots _1) \\&\text { if } \epsilon \in \mathcal {L}(M)\\ \circlearrowleft (\tau , \tau ) ={}&\tau \\ \circlearrowleft (\tau , \ldots _1) ={}&\circlearrowleft (\tau , a_1, \ldots , a_n) \\&\text { if } \{a_1 \ldots a_n\} = \varSigma (\ldots _1) \wedge \forall _{1 \le i \le n} \langle a_i \rangle \in \mathcal {L}(\ldots _1)\\&\text { and the resulting subtree is smaller than } \ldots _1 \\ \circlearrowleft (M, \tau ) ={}&\circlearrowleft (\tau , a_1, \ldots , a_n) \\&\text { if } \{a_1 \ldots a_n\} = \varSigma (M) \\&\quad \wedge \forall _{1 \le i \le n} \langle a_i \rangle \in \mathcal {L}(M) \wedge \epsilon \in \mathcal {L}(M)\\ \circlearrowleft (M, \tau ) ={}&\circlearrowleft (\times (a_1, \ldots , a_n), \tau ) \\&\text { if } \{a_1 \ldots a_n\} = \varSigma (M) \\&\quad \wedge \forall _{1 \le i \le n} \langle a_i \rangle \in \mathcal {L}(M) \wedge \epsilon \notin \mathcal {L}(M)\\&\text { and the resulting subtree is smaller than } M \end{aligned}$$

Obviously, each of these rules is language preserving and decreases the size of the process tree; the rules are applied exhaustively to both the projected system and the projected model.

## Appendix 3: Proof of Theorem 1

Theorem: Let *S* and *M* be process trees without duplicate activities and without $\tau$s. Then, ${\textit{recall}}(S, M, 2) = 1 \wedge {\textit{precision}}(S, M, 2) = 1 \Leftrightarrow \mathcal {L}(S) = \mathcal {L}(M)$.

### Proof

We prove the two cases $\Leftarrow$ and $\Rightarrow$ separately. Obviously, in both cases $\varSigma (S) = \varSigma (M)$. Take a set of activities $\{a, b\}$ with $\{a, b\} \subseteq \varSigma (M)$ and $a \ne b$.

- $\Leftarrow$ Assume $\mathcal {L}(S) = \mathcal {L}(M)$. Then obviously $\mathcal {L}(S|_{a, b}) = \mathcal {L}(M|_{a, b})$. By construction, $\text {DFA}(S|_{a, b}) = \text {DFA}(M|_{a, b})$ and hence
  ${\textit{recall}}(S, M, \{a, b\})$ = $precision(S, M, \{a, b\})$ = 1. This holds for all sets $\{a, b\})$; thus, recall and precision are 1.
- $\Rightarrow$ Assume ${\textit{recall}}(S, M, 2) = {\textit{precision}}(S, M, 2) = 1$. Then, ${\textit{recall}}(S, M, \{a, b\})$ = ${\textit{precision}}(S, M, \{a, b\}) = 1$, hence $\text {DFA}(S|_{a, b}) = \text {DFA}(M|_{a, b})$ and thus $\mathcal {L}(S|_{a, b}) = \mathcal {L}(M|_{a, b})$. By Corollary 15 in [29] and the assumed restrictions, for both *S* and *M*, there exist language-unique normal forms $S'$ and $M'$. Then, we may conclude that $\mathcal {L}(S'|_{a, b}) = \mathcal {L}(M'|_{a, b})$, and hence ,the lowest common parent of *a*, *b* in $S'$ is equal to the lowest common parent of $\{a, b\}$ in $M'$, and the relative order of *a* and *b* matches (in case of $\circlearrowleft$ or $\rightarrow$). This holds for all sets $\{a, b\}$, and neither tree contains duplicate activities or $\tau$s; thus, $S' = M'$, and hence, $\mathcal {L}(S) = \mathcal {L}(M)$. $\square$

## Appendix 4: Evaluation set-up details

In the experiments, directly-follows graphs were obtained using a small script that incrementally builds a directly-follows graph from an event log. If the experiment contained a memory limitation, this script was not exempt from that limitation.

We used ProM version 6.5.1a, and the logs are imported using the log importer ‘ProM log files (disk-buffered by MapDB, sequential access, w/o cache)’, as this importer requires the least amount of RAM. Enough SSD disk space was available for the importer.

Each experiment started with a new instance of the ProM framework, in order to release all memory.

For the log generation, whenever an $\times$-node was encountered, each child had an equal probability of being executed. For $\circlearrowleft$ nodes, the choice between doing a redo and an exit was a constant 0.5/0.5.

*Infrequent behaviour generation* For the infrequent behaviour experiment, we took the same log of $10^4$ activities generated from tree B, and added *n* deviating traces: for each trace, a random deliberate structural deviation was inserted at some point of execution, based on the operator on that point (each of these points with equal probability).

- $\times$ two children are executed,
- $\rightarrow$ either a child is executed a second time in an arbitrary position or a child is skipped,
- $\wedge$ either a child is executed twice or a child is not executed,
- $\circlearrowleft$ a child is skipped.

## Appendix 5: Projecting Petri nets and converting them into deterministic finite automata

A Petri net can be projected by replacing each transition that is not in *A* by a $\tau$-transition. In complex models and for smaller *k*s, the framework will introduce a lot of $\tau$-transitions in the projection. However, many of these $\tau$-transitions might be removable without changing the language of the projected model. Therefore, a subset of language-preserving reduction rules [39] can be applied to reduce the size of the Petri net, and hence its corresponding automaton.

A Petri net can be translated to an automaton using a state-space exploration. The more the net was reduced, the smaller the state space will be in this step.

A necessary condition for the translation to a DFA is that the language of the Petri net can be described by a DFA. A definition of a language requires the notion of start and acceptance of traces, and a finite state space, thus the model needs to be bounded, and provide an initial marking and a finite set of final markings. Please note that for general Petri nets, a translation to a DFA is impossible as we could use such a translation to decide language inclusion, which is undecidable [20]. Aware of this limitation, we introduce several heuristics, while making sure that for the consistency of the framework, the heuristics applied to a sound workflow net result in their normal semantics.

*Making a Petri net bounded* To make the Petri net bounded, each place is given an artificial capacity. During state-space exploration, a transition is only enabled if firing it would not violate the bound of any place [53]. As sound workflow nets are bounded, this heuristic will not influence their semantics. However, if the capacity is chosen too low, not enough behaviour might be captured for a comparison, and language-preserving reduction rules might influence the result. This limitation is inherent to using DFAs and solving it would require other classes of models, for which the problem might be undecidable.

*Heuristic for an initial marking* Some algorithms, such as the Heuristics Miner, provide an initial marking. If no initial marking is present, we construct an initial marking by putting a token into each place without incoming transitions. In sound workflow nets, an initial marking is provided by the source place, which corresponds to our heuristic.

*Heuristic for final markings* Only few discovery algorithms are capable of producing Petri nets with a distinguished final marking. In case no final marking is given, it can be obtained as follows:

- Manually inspect the model and define a final marking. This is usually infeasible for complex models;
- An approach taken in [3] is to consider each reachable marking as a final marking. Obviously, this increases the size of the language and is inconsistent with our framework: applying this heuristic to a sound workflow net results in an overestimation of recall and an underestimation of precision. Moreover, having each marking being a final marking is not what is *meant* by current discovery algorithms: corresponding to traces in the event log, algorithms aim to discover a model of a process with a clear start and a clear end. Nevertheless, in use cases in which such behaviour is intended [44], this strategy might be chosen (but must be chosen for both models to ensure comparability).
- Another approach is to consider each reachable conditional deadlock to be a final marking. A *conditional deadlock* is a marking *M* in which for each enabled transition *t*, one of the following conditions holds:
  - *t* is not connected to any place, or
  - firing *t* leads to a marking $M'$ that is equal to or strictly bigger than *M*.
  Figure 25 shows an example of a Petri net in a conditional deadlock: *b* is enabled but firing it would leave the net in a strictly larger marking (as *b* produces a token without consuming one), *d* is not connected to any place, and firing *e* would yield an equal marking. In sound workflow nets, the only conditional deadlock is the one with a token in the sink place, which corresponds to this heuristic. A downside of this strategy is that nets without conditional deadlocks (e.g. with livelocks) are considered to have the empty language.

We prefer the second strategy as it keeps the framework consistent and we performed the evaluation (Sect. 6) using it. Ideally, the discovery algorithm should provide an initial marking and final markings, and preferably a bounded net.

## Appendix 6: Evaluation process tree statistics

The table below gives for each process tree that was used in the evaluation its distribution of nodes.

**Table:**

	40 activities	1000 activities	10,000 activities
$\tau$	0	0	0
$\times$	3	142	1532
$\rightarrow$	6	212	2575
$\circlearrowleft$	2	86	1039
$\wedge$	6	130	1569

## Appendix 7: Discussion of evaluation results

### Scalability

For tree A, several implementations were limited by their requirement to load the log in main memory first: HM, IM, IMf, and IMc could handle $10^6$ traces, P-IF and P-PT $10^4$. The recursion on event logs of IM, IMf, and IMc shows its limitations as well. (Compared with [32], a more efficient log importer has been added to ProM, which enabled the import of $10^6$ traces; only HM benefits from this increase.) Only algorithms of the IMd framework could handle logs of $10^7$ or more traces, which shows the value of the single-pass property, as there is no need to load the log in main memory. IMd and IMfD are clearly not limited by log size. In [21], a single-pass version of HM and $\alpha$ is described. We believe such a version of HM could be memory-restricted, but still this would offer neither soundness nor rediscoverability. Of the IM framework, single-pass versions cannot exist due to the necessary log splitting. The exponential incompleteness-handling algorithms IMc and IMcD were unable to handle *smaller* logs (i.e. 1000 traces for IMc and 100 for IMcD); this has been denoted with a $\dagger$. These algorithms first try to find a perfect cut (like IM), and if this fails, i.e. for smaller logs, enter a procedure of exponential complexity.

Tree B challenged $\alpha$, HM, P-IF, P-PT, IMc, and IMcD more than tree A. Please note that HM handled the largest log we could generate, i.e. this experiment did not reach the limits of HM.

For tree C, log importers were not a problem. Algorithms that are exponential in the number of activities (IMc, IMcD, $\alpha$, HM) show their limitations here: none of them could handle a log with only 100 traces. Moreover, only IMd and IMfD could handle logs in which all 10,000 activities were present, which shows that in this case, sampling would not be an option: a log of a size manageable to other algorithms would not contain all activities (see Table 2). This experiment clearly shows the scalability of the IMd framework.

### Incompleteness

The flower models provide baselines for precision, i.e. 0.77 for model A and 0.66 for model B, and an upper bound for recall (1 for both), which depends on the completeness of activities in the event log.

HM illustrates the challenges that arise when dealing with arbitrary Petri nets. As HM does not provide a final marking, and the models contained no obvious final places (and our heuristic (Appendix 5) could not find a final marking either), the languages of these models remained unclear and we didn’t include their results (recall 0, precision 1).

For model A, IM and IMd performed similarly: both achieved perfect recall at 10 traces (the first time all activities had appeared in the event log), and both reached perfect precision at $10^4$. Manual inspection revealed that IMd and IM always returned the same model once the log was complete. The infrequent-handling IMf and IMfD performed similar to IM and IMd, except for $10^3$ traces, where recall dropped and precision remained comparable. Manual inspection revealed that the root operator of A is $\wedge$; at $10^2$ traces, both miners did not find a cut and returned general models ($\circlearrowleft (\ldots ,\tau ,\tau )$) yielding a high recall, whereas at $10^3$ traces, the miners found $\wedge$-cuts but got the activity partition wrong.

For model B, IMd and IM performed similarly: both reached perfect recall at $10^5$ (once the log contained all activities), while IMd achieved a slightly higher precision for each log in this experiment. At $10^8$, IMd reached perfect precision, while IM could not handle logs of this size. The results for IMfD and IMf contain a drop in recall at $10^4$ traces (just as for tree A) and show the same precision as IMd and IM. Given the results presented in [30], IMc and presumably IMcD would have performed better in these experiments. However, as shown by the scalability results, IMc and a corresponding IMcD are unable to handle logs of these sizes.

### Infrequent Behaviour

Figure 20 shows the results for the infrequency experiment. Similar to the noise experiments, the infrequency-filtering algorithms (IMf, IMfD) perform slightly better than IM and IMd. Except for $n = 10^5$, IMfD got the highest precision scores for each *n*. Similarly, IMd got a consistently higher precision than IMfD. As the event logs were equivalent (up to the introduction of noise/infrequent behaviour) for the noise experiment and this experiment, we expected a similar increase in precision for IMd at $n = 10^5$. However, it was IMf that got this increase; IMd and IM remained at their lower level.

Nevertheless, IMd and IMfD seem to do better than IM and IMf on precision, which seems counterintuitive as the IM framework can use more information than the IMd framework. We suspect that two of the inserted types of infrequent behaviour might be of influence here: skipping a child of a $\rightarrow$ or $\wedge$ has no troublesome impact on the directly-follows graph for the IM framework, but log splitting will introduce a (false) empty trace for the IM framework; IM framework algorithms must decide to ignore this empty trace in later recursions, while IMd framework algorithms simply don’t see it.

Altogether, IMfD performs remarkably well and stable in this typical case of process discovery where an event log contains structured deviations from the system.

## Appendix 8: Evaluation results

### Incompleteness

See Figs. 26 and 27. The models for IMc for $10^3$ traces and IMcD for $10^2$ traces could not be obtained (they ran for over a week).

### Noise

See Fig. 28.

### Infrequent behaviour

See Fig. 29.

## Footnotes

1 Available for download at http://promtools.org.

2 Anecdotically: the vector images of these models were too large to be displayed by Adobe Illustrator or Adobe Acrobat.
